# Syntrophic Acetate-Oxidizing Microbial Consortia Enriched from Full-Scale Mesophilic Food Waste Anaerobic Digesters Showing High Biodiversity and Functional Redundancy

**DOI:** 10.1128/msystems.00339-22

**Published:** 2022-09-08

**Authors:** Chao Li, Liping Hao, Fan Lü, Haowen Duan, Hua Zhang, Pinjing He

**Affiliations:** a Institute of Waste Treatment and Reclamation, Tongji University, Shanghai, People’s Republic of China; b State Key Laboratory of Pollution Control and Resource Reuse, College of Environmental Science and Engineering, Tongji University, Shanghai, People’s Republic of China; c Shanghai Institute of Pollution Control and Ecological Security, Shanghai, People’s Republic of China; Los Alamos National Laboratory

**Keywords:** anaerobic digestion, syntrophic acetate oxidation, methanogenic pathway, metagenomic analysis, biodiversity, functional redundancy

## Abstract

Syntrophic acetate oxidation (SAO) coupled with hydrogenotrophic methanogenesis (HM) plays a vital role in the anaerobic digestion of protein-rich feedstocks such as food wastes. However, current knowledge of the biodiversity and genetic potential of the involved microbial participants, especially syntrophic acetate-oxidizing bacteria (SAOB), is limited due to the low abundance of these microorganisms and challenges in their isolation. The intent of this study was to enrich and identify potential SAOB. Therefore, we conducted continuous acetate feeding under high ammonia concentrations using two separate inoculum consortia of microorganisms that originated from full-scale mesophilic food waste digesters, which lasted for more than 200 days. Using 16S rRNA gene amplicon and metagenomic analyses, we observed a convergence of the experimental microbial communities during the enrichment regarding taxonomic composition and metabolic functional composition. Stable carbon isotope analyses of biogas indicated that SAO-HM was the dominant methanogenic pathway during the enrichment process. The hydrogenotrophic methanogen *Methanoculleus* dominated the archaeal community. The enriched SAO community featured high biodiversity and metabolic functional redundancy. By analyzing the metagenome-assembled genomes, the known SAOB Syntrophaceticus schinkii and six uncultured populations were identified to have the genetic potential to perform SAO through the conventional reversed Wood-Ljungdahl pathway, while another six bacteria were found to encode the reversed Wood-Ljungdahl pathway combined with a glycine cleavage system as novel SAOB candidates. These results showed that the food waste anaerobic digesters harbor diverse SAOB and highlighted the importance of the glycine cleavage system for acetate oxidation.

**IMPORTANCE** Syntrophic acetate oxidation to CO_2_ and H_2_, together with hydrogenotrophic methanogenesis, contributes to much of the carbon flux in the anaerobic digestion of organic wastes, especially at high ammonia concentrations. A deep understanding of the biodiversity, metabolic genetic potential, and ecology of the SAO community can help to improve biomethane production from wastes for clean energy production. Here, we enriched the SAO-HM functional guild obtained from full-scale food waste anaerobic digesters and recorded dynamic changes in community taxonomic composition and functional profiles. By reconstructing the metabolic pathways, diverse known and novel bacterial members were found to have SAO potential via the reversed Wood-Ljungdahl (WL) pathway alone, or via the reversed WL pathway with a glycine cleavage system (WLP-GCS), and those catalyzing WLP-GCS showed higher microbial abundance. This study revealed the biodiversity and metabolic functional redundancy of SAOB in full-scale anaerobic digester systems and provided inspiration for further genome-centric studies.

## INTRODUCTION

Anaerobic digestion has been widely used to treat wastewater and organic waste and to recover renewable energy (in the form of biogas or biomethane as fuel) and resources (in the form of nutrient-rich digestate as soil fertilizer). Acetate is the most important methanogenic precursor during the digestion process, since it has been predicted that 60 to 80% of methane is produced from acetate (1). Acetate can be converted into methane and carbon dioxide either directly by acetotrophic methanogens (e.g., *Methanothrix*) or through a two-step reaction in which it is first oxidized to carbon dioxide and hydrogen by syntrophic acetate oxidizing bacteria (SAOB), and then hydrogenotrophic methanogens (e.g., *Methanoculleus*) reduce carbon dioxide to methane with hydrogen as an electron donor (2, 3). Protein-rich substrates such as chicken manure and food waste (FW) are common feedstocks in biogas plants due to their high biomethane potential. However, simultaneous risks exist, since substantial amounts of ammonia are produced during the digestion of these substrates, which can inhibit the metabolic activity of microorganisms and even cause failure of the system (4, 5). Compared with hydrogenotrophic methanogens, acetotrophic methanogens are more sensitive to ammonia, and thus syntrophic acetate oxidation (SAO) coupled with the hydrogenotrophic methanogenesis (SAO-HM) pathway would replace the acetoclastic methanogenesis (AM) pathway and become dominant under ammonia-inhibited conditions (6, 7).

Although SAOB are especially important for the stable performance of anaerobic digesters, they are typically low in abundance and difficult to isolate (8); thus, current knowledge about their diversity and genetic potential is limited. Two metabolic SAO pathways have been proposed: the conventional reversed Wood-Ljungdahl (WL) pathway and a novel metabolism combining the reversed WL pathway with a glycine cleavage system (WLP-GCS) (9). The reversed WL pathway is frequently found in known SAOB, including Syntrophaceticus schinkii (10), Tepidanaerobacter acetatoxydans (11), and Thermacetogenium phaeum (12). However, the relative abundance of these few known SAOB is usually very low in anaerobic digesters (13), indicating that there might be other uncultured syntrophic bacteria that can oxidize acetate but are not yet recognized. Recent studies have indicated that some bacteria have the genetic potential to convert acetate to H_2_ and CO_2_ via the WLP-GCS pathway (14, 15), which notably increases the diversity of potential SAOB candidates and points to a yet-to-be revealed functional redundancy of this metabolic capability in the microbial community. Several studies have been dedicated to SAOB in anaerobic digesters, and a few novel organisms have been identified. For example, uncultured bacteria such as *Synergistes* group 4 (16), subspecies of *Clostridia* (17), unFirm_1, which showed 88% similarity to Pelotomaculum thermopropionicum (18), members within the family *Clostridiales* (19) and the genera *Coprothermobacter*, *Mesotoga*, *Aminivibrio*, *Acetivibrio*, *Desulfovibrio*, *Petrimonas*, *Sedimentibacter*, and several unclassified bacteria (20–22).

SAOB may play a key role in FW anaerobic digestion. Our previous work illustrated the significant contribution of SAO-HM in mesophilic FW digesters and the potential existence of such a functional guild in the microbiota (23). The taxonomic and genetic diversity of the SAO community is, however, not clear, although novel SAOB candidates, such as species of *Acetomicrobium*, *Thermoanaerobacteraceae*, and *Clostridia*, were shown to appear in thermophilic (53 to 55°C) FW digesters (24, 25). As temperature has a significant impact on the thermodynamics of SAO-HM reactions and the life cycle of SAOB and methanogens (13), the composition of the functional guild and behavior of the members under mesophilic conditions can be considerably different from those under thermophilic conditions. Nearly 200 full-scale FW anaerobic treatment facilities have been built in China, most of which are operated under mesophilic conditions (35 to 40°C). It is therefore important to reveal the presence of potential SAOB and identify their role in these mesophilic digesters.

Continuous feeding with volatile fatty acids (VFAs) is known to enrich syntrophic VFA-oxidizing bacteria (19, 26). The combination of this enrichment process with high-throughput metagenomic sequencing makes it possible to acquire high-quality draft genomes of some novel syntrophic members with low abundance in full-scale digesters (27). Previous studies on this topic have focused on the culminating enriched microbiota (25, 26), but less attention has been given to the dynamics of anaerobic microbial communities and metabolic pathways that could be enriched as important to understand how beneficial microbial consortia gradually evolve to a relatively stable status.

In this study, syntrophic acetate-oxidizing microbial communities were enriched from separate microbiota obtained from two mesophilic full-scale anaerobic digesters named as CZ and LM, respectively. Both digesters were treating food waste, but illustrated low and high contributions of the SAO-HM pathway, respectively (23). The enrichment process was performed for more than 200 days by continuously feeding the experimental microbial communities with acetate. During this process, the microbial community structure and the methanogenic pathway were dynamically monitored. The key microbial players and their genetic potential in acetate metabolism were studied by metagenomic binning and genome annotation before and after enrichment, which ultimately resulted in the identification of several novel SAOB candidates.

## RESULTS AND DISCUSSION

### Reactor performance during the enrichment process.

**FIG 1:**
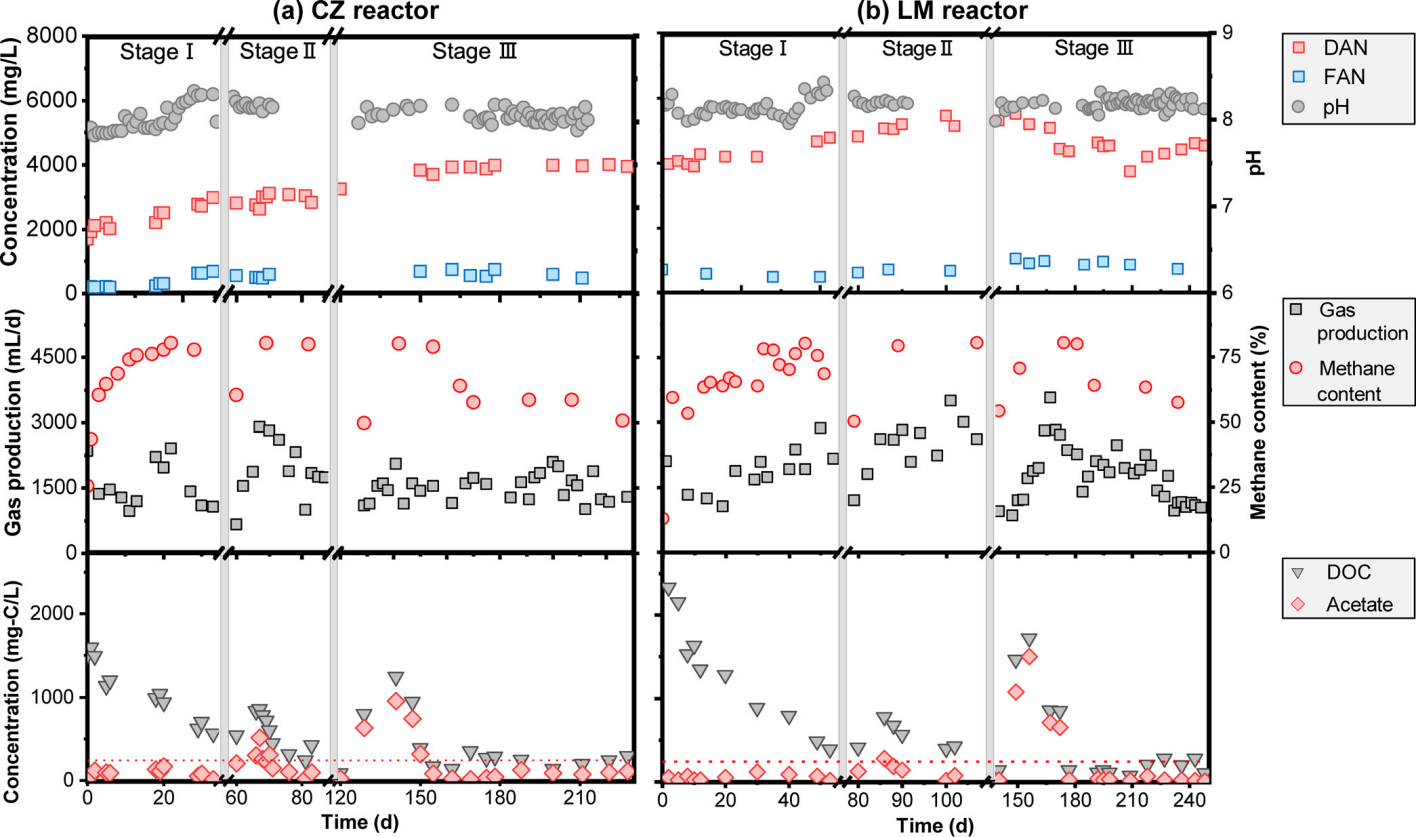
Reactor performance during the enrichment process for the CZ reactor (a) and LM reactor (b), represented by concentrations of dissolved ammonium nitrogen (DAN), free ammonia nitrogen (FAN), and pH; gas production and methane content; and concentrations of dissolved organic carbon (DOC) and acetate. The dashed red line indicates the added acetate concentration by the daily feedstock (240 mg of C/L).

The microbiota taken from full-scale CZ and LM digesters were respectively used as inoculum for the lab-scale CZ and LM reactors. Before being cultivated, they lived in relatively different environments, as the LM full-scale digester demonstrated higher levels of pH, concentration of dissolved organic carbon (DOC), dissolved ammonium nitrogen (DAN), free ammonia nitrogen (FAN), and VFAs than those in the CZ full-scale digester (see Fig. S1 in the supplemental material), indicating higher environmental pressure generated from these factors. After being cultivated, these factors in the lab-scale CZ and LM reactors for cultivation were set at similar levels but demonstrated gradual elevation and slight fluctuation. During the enrichment process, the pH values fluctuated between 7.8 and 8.4 in the CZ reactor and between 8.0 and 8.4 in the LM reactor (Fig. 1). Due to the introduction of extra ammonium from the feedstock, the NH_4_^+^ concentrations in both reactors rose gradually with enrichment time, reaching 3,923 ± 54 mg/L in the CZ reactor and 4,379 ± 265 mg/L in the LM reactor in stage III. Correspondingly, the FAN concentrations in the same period were 616 ± 97 mg/L in the CZ reactor and 798 ± 95 mg/L in the LM reactor and were dramatically affected by pH (8.11 ± 0.08 in the CZ reactor and 8.19 ± 0.07 in the LM reactor). Biogas production varied over a wide range between 0.66 and 2.90 L/day in the CZ reactor and 0.84 to 3.56 L/day in the LM reactor, with the methane contents varying between 50 and 80%. The two operational gaps resulted in two starvation perturbations to the systems, which might be responsible for the fluctuation of the reactor performance when restarted (28). Acetate was observed to accumulate in the beginning of stage II (as high as 512 mg of C/L in the CZ reactor and 277 mg of C/L in the LM reactor) and stage III (950 mg of C/L in the CZ reactor and 1,504 mg of C/L in the LM reactor), but in the following 20 to 36 days, it gradually decreased to levels similar to those before the perturbation (3.07 ± 1.78 mM in the CZ reactor and 1.61 ± 1.20 mM in the LM reactor).

10.1128/msystems.00339-22.5 FIG S1 Comparison of physicochemical properties and microbial diversities (based on amplicon data analysis) of full-scale anaerobic digesters where the inoculating microbiota come from. Download FIG S1, PDF file, 0.3 MB. Copyright © 2022 Li et al. 2022 Li et al. https://creativecommons.org/licenses/by/4.0/ This content is distributed under the terms of the Creative Commons Attribution 4.0 International license.

### Evolution of microbial communities during the enrichment process.

**FIG 2:**
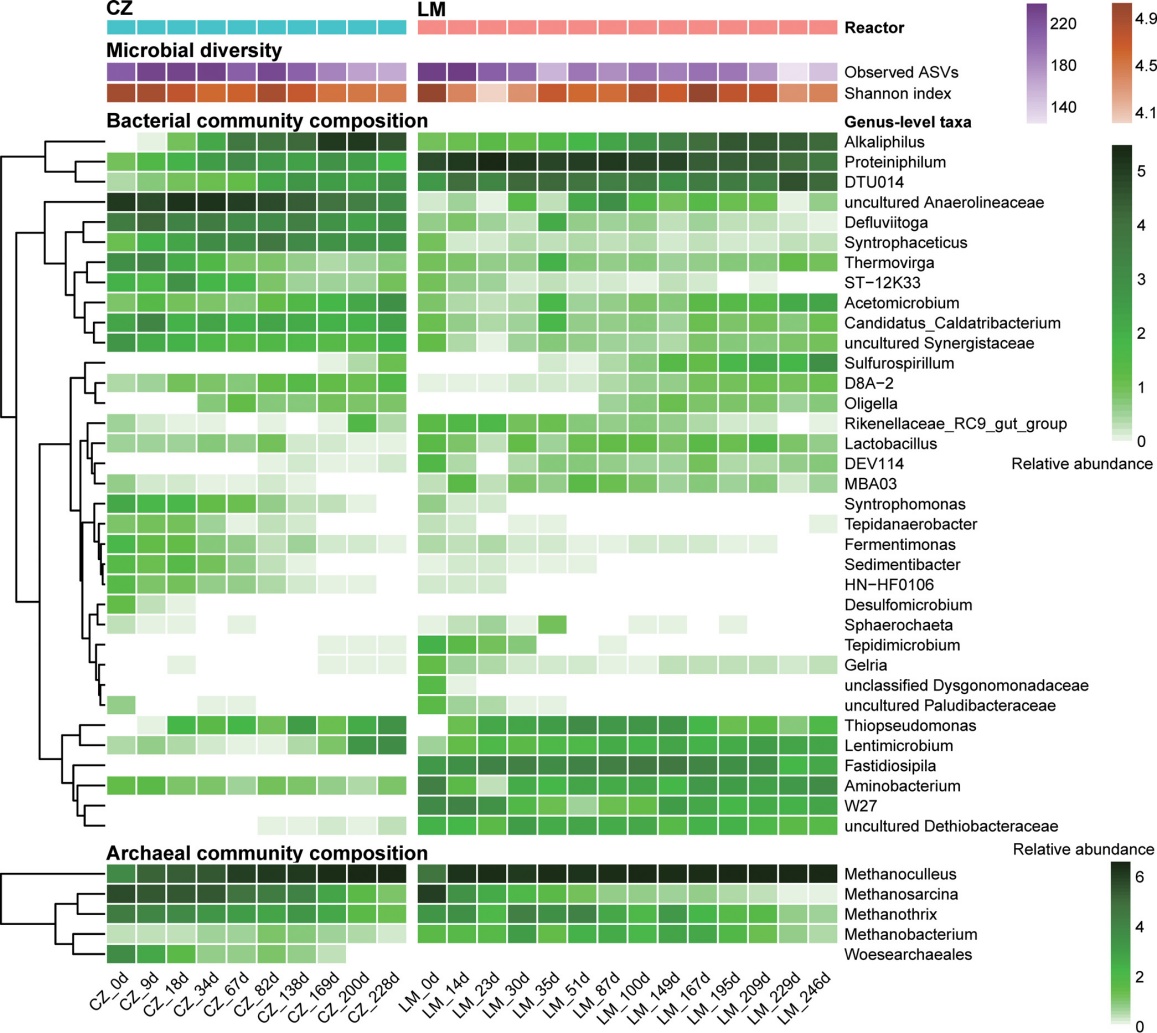
Dynamic changes of microbial community diversity and composition in CZ and LM reactors during the enrichment process, determined by 16S rRNA gene amplicon sequencing. Genus-level taxa with any occurrence of >1% are shown. Relative abundance was calculated as log_2_(reads percentage + 1) for visualization, which was also applied to Fig. 5 and Fig. 6.

During the enrichment process, dynamic changes in the microbial community composition along with enrichment time were analyzed by using 16S rRNA gene amplicon sequencing. The microbial community structure in both reactors changed greatly from the inoculating microbiota in the first 2 weeks (see Fig. S2). The alpha diversity of the microbial communities illustrated a decreasing trend during the enrichment process, including the number of observed ASVs and the Shannon index (Fig. 2).

10.1128/msystems.00339-22.6 FIG S2 Area chart representing the relative abundance of the top 12 bacterial phyla (based on amplicon data analysis) in a CZ reactor (a) and an LM reactor (b) during the enrichment process. (c) Dynamic change of compositional dissimilarity of the microbial communities (based on amplicon data analysis) in a CZ reactor and an LM reactor. Amount of hydrogen in the collected biogas during the enrichment process in a CZ reactor (d) and an LM reactor (e). Download FIG S2, PDF file, 0.2 MB. Copyright © 2022 Li et al. 2022 Li et al. https://creativecommons.org/licenses/by/4.0/ This content is distributed under the terms of the Creative Commons Attribution 4.0 International license.

The relative abundance of the phylum *Firmicutes* gradually increased and became the most abundant phylum in each of the cultivated systems (see Fig. S2). In contrast, the phylum *Chloroflexi* in the CZ reactor and *Bacteroidota* in the LM reactor gradually decreased with enrichment time. When we looked at the genus level, we found that five abundant genera (with relative abundances of >1% of the total bacterial sequences in at least one sample) significantly increased (*P < *0.05) in relative abundance with time in both reactors (see Fig. S3), namely, *Acetomicrobium*, *Alkaliphilus*, D8A-2, *Lentimicrobium*, and *Sulfurospirillum*. In addition, DEV114, DTU014, *Tepidimicrobium*, *Thiopseudomonas*, and uncultured *Dethiobacteraceae* in the CZ reactor and *Oligella* in the LM reactor also increased in abundance during the enrichment process.

10.1128/msystems.00339-22.7 FIG S3 (a) Dynamic changes of relative abundance of selected bacterial genera in the two lab-scale reactors. (b) Spearman’s correlations between the *Methanoculleus* and bacterial members *Alkaliphilus*, D8A-2, *Oligella*, and *Acetomicrobium* based on amplicon data analysis. Download FIG S3, PDF file, 0.5 MB. Copyright © 2022 Li et al. 2022 Li et al. https://creativecommons.org/licenses/by/4.0/ This content is distributed under the terms of the Creative Commons Attribution 4.0 International license.

The relative abundance of the hydrogenotrophic methanogen *Methanoculleus* increased rapidly during the process, reaching 97 to 98% of the archaeal community in both reactors after enrichment (Fig. 2). Spearman’s correlation analysis (see Fig. S3) revealed highly positive correlations between *Methanoculleus* and the bacterial members *Acetomicrobium*, D8A-2, *Alkaliphilus*, and *Oligella*, indicating that these members played essential roles in acetate-fed systems.

**FIG 3:**
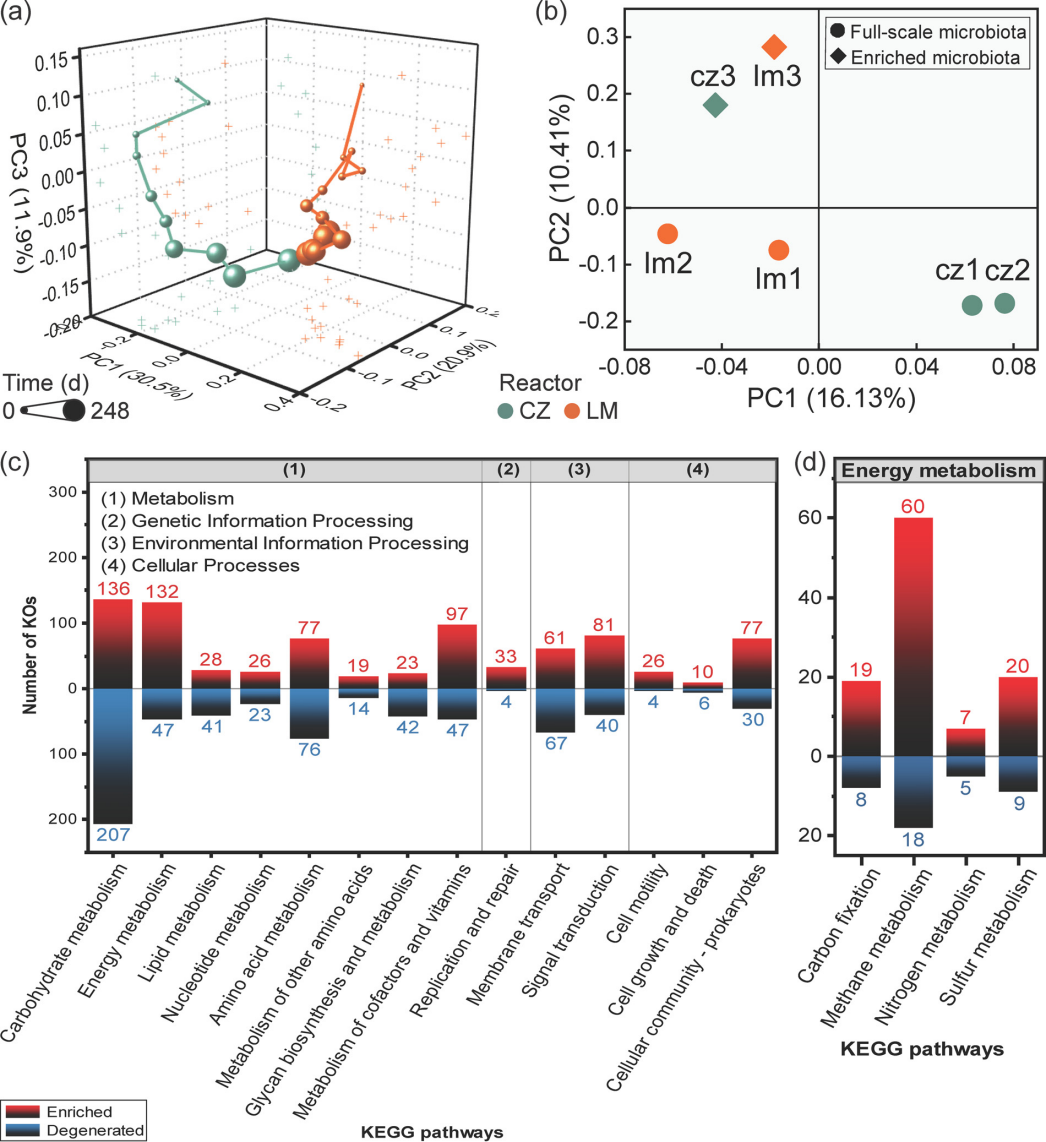
PCoA of microbial community structure derived from 16S rRNA gene analysis using weighted UniFrac distance (a) and functional composition derived from metagenomic analysis using Bray-Curtis distance (b). Numbers of KEGG orthogroups enriched (after/before ratio > 2.0) or degenerated (after/before ratio < 0.5) during the enrichment process (c) and KEGG orthogroups specifically participated in energy metabolism (d). The six metagenomes were obtained by sequencing samples taken 2 months before (CZ1 and LM1, from the full-scale digesters), just at the start (CZ2 and LM2, from the full-scale digesters), and after (CZ3 and LM3, from the lab-scale reactors) the enrichment process. Functional category information was obtained by referring to the KEGG BRITE database.

Although the community structure of the inoculating microbiota differed significantly between the two systems based on clustering and principal coordinate analysis (PCoA) results (see Fig. S4), they converged gradually during the enrichment process (Fig. 3a) under the similar cultivating conditions (i.e., same carbon and nitrogen sources, similar pH levels, and ammonia stress). This result indicated that deterministic mechanisms guided the succession of the microbial communities during this enrichment process (29).

10.1128/msystems.00339-22.8 FIG S4 Comparison of bacterial (a) and archaeal (b) communities in full-scale anaerobic digesters where the inoculating microbiota come from. (c) PCoA of microbial community structure (based on amplicon data analysis) of full-scale anaerobic digesters where the inoculating microbiota come from. Download FIG S4, PDF file, 0.2 MB. Copyright © 2022 Li et al. 2022 Li et al. https://creativecommons.org/licenses/by/4.0/ This content is distributed under the terms of the Creative Commons Attribution 4.0 International license.

### Overall shifts in functional genes and KEGG pathways during the enrichment process.

Information on 5,701 distinct genes with K numbers annotated by Kyoto Encyclopedia of Genes and Genomes (KEGG) orthology (KO) were extracted from six metagenomes. The PCoA result based on the relative abundances of these genes is shown in Fig. 3b. The two samples taken before and just at the start of the enrichment process from the same reactor were in the same cluster, forming a CZ cluster (CZ1 and CZ2) and an LM cluster (LM1 and LM2), which were different from each other. After enrichment, samples shifted far away from their original cluster before enrichment and formed a new cluster (CZ3 and LM3). The discrepancy in functional profiles between different digester samples decreased drastically during the experiment, which indicated a convergence trend of the community functional composition.

A total of 1,217 genes represented by different K numbers were found to be enriched during the process, with an after/before ratio of >2 in both reactors, while 910 genes decreased in abundance, with an after/before ratio of <0.5. The functional categories of these genes were identified by referring to the KEGG BRITE database. As shown in Fig. 3c, at the highest BRITE hierarchy level, genes related to energy metabolism illustrated a notable increase after enrichment, in which 132 KOs were enriched while only 47 were reduced in abundance. A further look into the subcategories of energy metabolism revealed that most of the KOs involved in methane metabolism, carbon fixation, and sulfur metabolism were enriched (Fig. 3d), among which the methanogenesis and reversed WL pathways were included. The enrichment of genes involved in sulfur metabolism could be related to the enriched *Sulfurospirillum* members during the process (Fig. 2; see also Fig. S4), which can oxidize sulfide (30). In addition, many more genes involved in cofactor and vitamin metabolism were enriched than those that decreased (97 versus 47), indicating that the enriched microbiota might need more cofactors and vitamins for the metabolic activities required for turning acetate to methane, as was different from that in full-scale digesters. In addition, BRITE genetic information processing, such as replication and repair; environmental information processing, such as signal transduction; and cellular processes, such as cellular community, cell motility and cell growth and death, were all enriched, indicating the active growth of new cells. In contrast, most of the genes involved in carbohydrate metabolism, lipid metabolism, and glycan biosynthesis and metabolism were reduced, which could be due to a lack of required substrates.

In addition to the overall genetic potential of the whole communities, we also studied individual microbial members by annotating the metagenome-assembled genomes (MAGs), with 1,534 nonredundant MAGs obtained from the six samples. The proportion of metagenomic reads that could be mapped to these MAGs ranged from 72.0 to 74.0%. Furthermore, 567 of these MAGs were successfully taxonomically classified with gtdbtk, accounting for 80.2 to 92.0% of the reads mapped to these 1,534 MAGs.

Microbial taxonomic composition at the phylum level for the six samples based on MAG annotation was compared to that based on 16S rRNA gene amplicon annotation (see Fig. S5). *Firmicutes* was identified as the most abundant phylum using both annotation methods, contributing to 23.3% of the metagenomic reads and 24.2% of the 16S rRNA gene amplicon sequences. The phyla *Bacteroidota*, *Cloacimonadota*, and *Proteobacteria* also demonstrated similar abundances with both methods, but the other phyla demonstrated some discrepancies, such as *Chloroflexota* (6.2% by amplicon sequencing versus 19.4% by metagenomic sequencing), *Halobacteriota* (20.9% versus 9.6%), *Thermotogota* (12.7% versus 0.6%), and *Synergistota* (10.6% versus 2.2%), without considering the influence of the unclassified MAGs (14.1%).

10.1128/msystems.00339-22.9 FIG S5 Comparison of microbial composition based on taxonomic annotation of MAGs (inner ring) and 16S rRNA gene amplicons (outer ring) on the phylum level for the six samples. Download FIG S5, PDF file, 0.2 MB. Copyright © 2022 Li et al. 2022 Li et al. https://creativecommons.org/licenses/by/4.0/ This content is distributed under the terms of the Creative Commons Attribution 4.0 International license.

The quality of the 1,534 MAGs was assessed. Among them, 156 MAGs illustrated a completeness of >80% and contamination of <5% and were determined to be medium- to high-quality MAGs in this study. These MAGs included 146 bacterial members and 10 archaeal members, accounting for 40.4 to 58.5% of the DNA sequences in each metagenomic data set and were used for downstream analyses to identify the functional microbial members involved in methane production (see Data Set S1).

10.1128/msystems.00339-22.1 DATA SET S1 (a) Description of the full-scale food waste anaerobic digesters, where the inoculating microbiota comes from. (b) Composition of the modified basal medium. (c) Composition of the stock trace element solution and the vitamin solution in the modified basal medium. (d) Basic information on the eight sets of metagenomic bins. (e) Sequencing strategy of slurry samples for DNA sequence analysis. Download Data Set S1, XLSX file, 0.07 MB. Copyright © 2022 Li et al. 2022 Li et al. https://creativecommons.org/licenses/by/4.0/ This content is distributed under the terms of the Creative Commons Attribution 4.0 International license.

### Methane production metabolism and methanogens.

**FIG 4:**
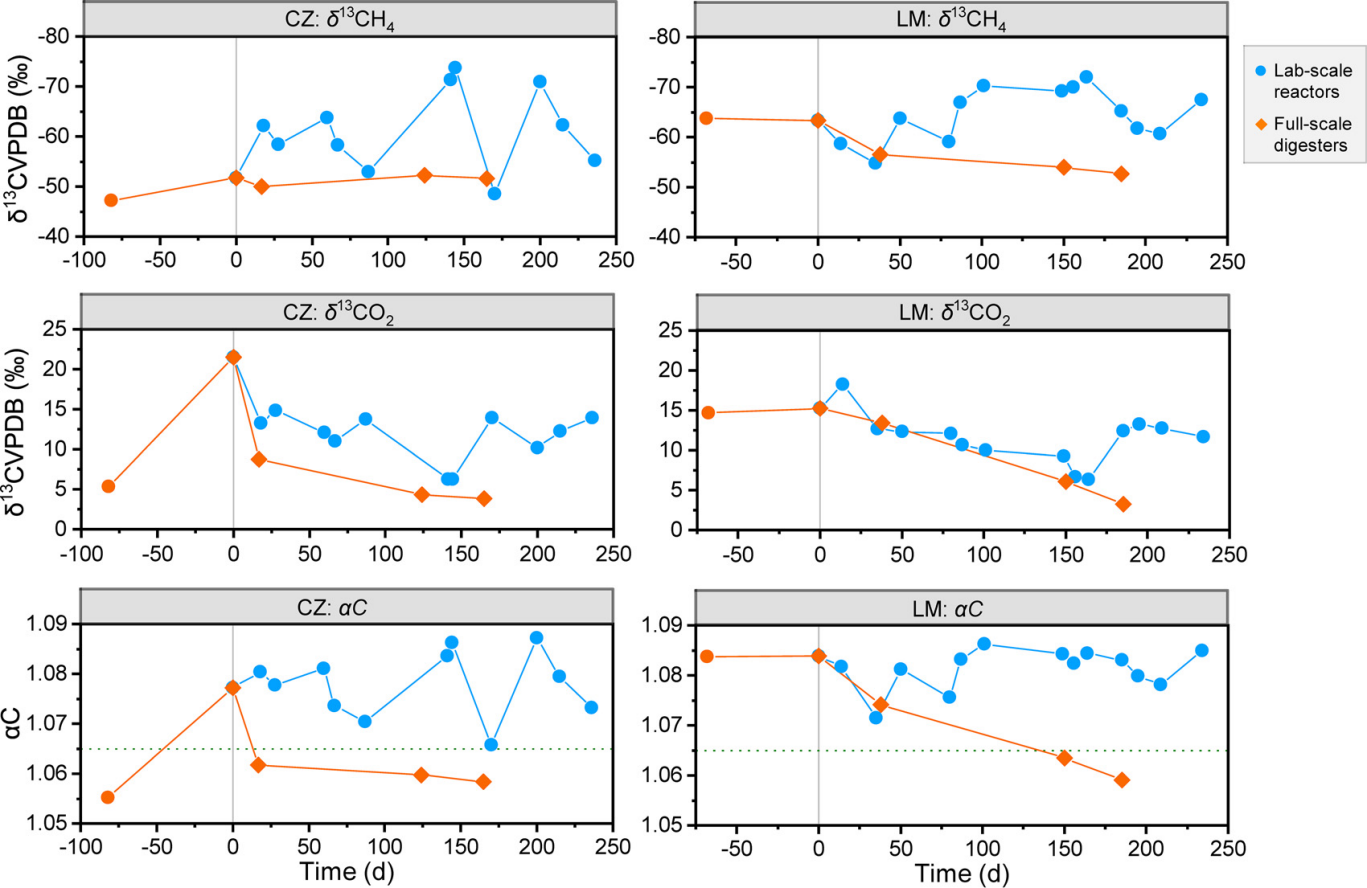
Simultaneous monitoring of stable carbon isotope signature of biogas in lab-scale acetate-fed reactors and full-scale food waste digesters providing the inoculating microbiota at the same period. A green dotted line was added to highlight the specific value of α_C_ (1.065) to distinguish the predominance of hydrogenotrophic methanogenesis. Day 0 represented initiation of the lab-scale reactors, and the biogas samples taken together with the inoculating microbiota from the full-scale digesters on that date were analyzed.

The dominant pathway of methanogenesis can be evaluated by analyzing the stable carbon isotope composition of CH_4_ (δ^13^CH_4_) and CO_2_ (δ^13^CO_2_) in the biogas and by calculating the ^13^C apparent fractionation factor (α_C_). The hydrogenotrophic pathway leads to a stronger stable carbon isotope fractionation effect than the acetoclastic pathway, as demonstrated by lower δ^13^CH_4_ values and higher α_C_ values. Generally, α_C_ > 1.065 indicates a predominance of HM, while α_C_ < 1.055 indicates a higher contribution of AM (31). The variations in *δ*^13^CH_4_ and *δ*^13^CO_2_ in lab-scale reactors during the enrichment process and in full-scale digesters during the same period were monitored, and *α*_C_ was calculated. As shown in Fig. 4, δ^13^CH_4_ and δ^13^CO_2_ of the lab-scale reactor samples fluctuated in the ranges of −48.6 to −73.7‰ and 6.3 to 21.5‰, respectively, and the α_C_ values fluctuated between 1.065 and 1.088 (1.078 ± 0.006 in the CZ reactor and 1.081 ± 0.004 in the LM reactor) during the enrichment process. The α_C_ value at the beginning of the enrichment (1.075) was already at a relatively elevated level (32), indicating the predominance of HM, which showed inhibition of AM in the CZ system but was a continuation in the LM system. Nevertheless, the α_C_ value has been reported to differ even in microbial cultures that sustain a defined chemical reaction (31). Stronger stable carbon isotope fractionation effects were found in the laboratory reactors than in the full-scale digesters, still indicating changes in biochemical reactions and microbial compositions.

**FIG 5:**
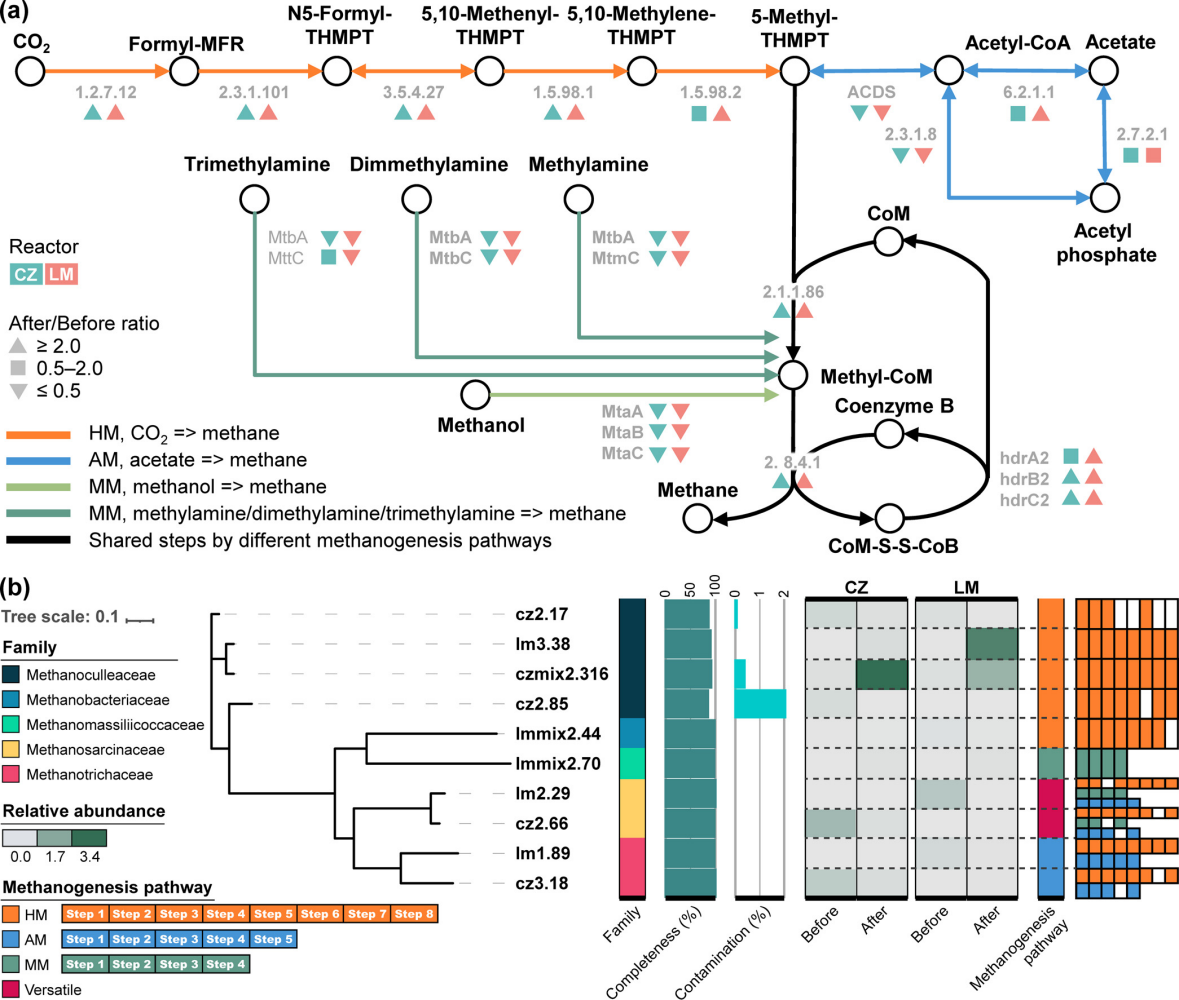
(a) Methanogenic pathways detected in the metagenomes and the abundance change of enzyme-encoding genes before and after the enrichment (represented by the after/before ratio). For enzyme encoded by more than one gene, it was denoted as colored triangles if the after/before ratio of all corresponding essential genes were >2; but as long as the after/before ratio of one essential gene was <0.5, it was labeled with an inverted color triangle. Colored boxes are used in cases that are different from the aforementioned two situations (details can be found in Data Set S3). (b) Phylogenetic tree of 10 archaeal MAGs (completeness > 80% and contamination < 5%). The relative abundance of each MAG in the two digester groups, before and after the enrichment, is shown in the heatmap. Existence data for genes associated with different methanogenic pathways in these MAGs are shown as colored rectangles.

Variations in functional genes participating in different methanogenic pathways are shown in Fig. 5a, represented by the After/Before ratio of relative read abundance. The genes in the HM pathway (such as *mch*, *ftr*, and *mer*) increased in relative abundance after enrichment, together with the genes shared by all three methanogenic pathways. In comparison, genes indispensable for the acetoclastic pathway (such as *cdhCDE*) or methylotrophic methanogenesis (such as *mta* and *mtb*) decreased markedly in relative abundance. Such a change in the genetic composition was consistent with the change in the dominant pathway indicated by the carbon isotopic signature and the methanogenic archaeal populations: the hydrogenotrophic *Methanoculleus* became dominant, while the acetotrophic *Methanothrix* and *Methanosarcina* gradually decreased.

10.1128/msystems.00339-22.3 DATA SET S3 Relative abundance of genes participated in degradation of acetate or production of methane in six samples and the presence or absence of these key genes in potential SAOB or methanogens. Download Data Set S3, XLSX file, 0.7 MB. Copyright © 2022 Li et al. 2022 Li et al. https://creativecommons.org/licenses/by/4.0/ This content is distributed under the terms of the Creative Commons Attribution 4.0 International license.

Regarding the individual MAGs contributing to methanogenesis, MAGs lm3.38 and czmix2.316 were annotated as *Methanoculleus*, which encoded all the functional genes directly involved in HM (Fig. 5b). After enrichment, the relative abundance of lm3.38 increased up to 182-fold (0.03 to 6.10%) in the LM reactor and 55-fold (<0.01 to 0.20%) in the CZ reactor, and that of czmix2.316 also increased by 77 times (0.12 to 9.61%) in the CZ reactor and 13 times (0.12 to 1.68%) in the LM reactor. Another two MAGs were also identified as hydrogenotrophic methanogens, with cz2.85 identified as *Methanoculleus* and lmmix2.44 assigned to *Methanobacteriaceae*, but their relative abundance in the cultivated system was low (<0.05%).

MAGs lm1.89 and cz3.18 were classified as *Methanothrix*. In both draft genomes, nearly all functional genes directly involved in the AM pathway were found, except for the *mcr* gene, which was missing in MAG cz3.18. Surprisingly, both acetotrophic members encoded a complete or nearly complete list of genes involved in the reduction of carbon dioxide to methane, indicating genetic potential for directly utilizing H_2_ or extracellular electrons with other partners, such as *Geobacter* (33), which was nevertheless lacking in the current systems. MAGs cz2.66 and lm2.29 were classified as *Methanosarcina*, which encoded a complete or nearly complete list of genes necessary for all three methanogenic pathways, showing versatile metabolic genetic potential. However, neither *Methanothrix* nor *Methanosarcina* members seemed to be actively working during the enrichment, as all four methanogenic populations experienced a sharp decrease in relative abundance (see Data Set S1).

Except for the hydrogenotrophic and acetotrophic populations, some methylotrophic methanogens (2) were also enriched but in relatively low abundance. For example, MAG lmmix2.70 was assigned to the family *Methanomassiliicoccaceae* and possessed all the genes directly involved in the methylotrophic methanogenesis (MM) pathway using methanol or methylamine/dimethylamine/trimethylamine as the substrate and H_2_ as the electron donors, indicating genetic potential for utilizing methylated compounds (possibly from decay of cells) and hydrogen for energy metabolism. The relative abundance of this member increased from 0 to 0.02% in full-scale digesters to 0.1% after enrichment in both lab-scale reactors, indicating that it was active. Members within *Methanomassiliicoccaceae* were observed to assimilate carbon from acetate in a previous study (34) and were predicted to utilize acetate as the carbon source for anabolism (35). In MAG lmmix2.70, we indeed found the acetyl coenzyme A (acetyl-CoA) synthetase gene, which supported the assumption and possibly explained why such methylotrophic members could also be enriched with acetate.

The detection of hydrogen in the exported biogas (see Fig. S2) indicated that hydrogen was actively produced from the added acetate, providing a substantial precursor for hydrogenotrophic methanogenesis. Thus, there must be some microbial players catalyzing the conversion of acetic acid to hydrogen and CO_2_, since acetate was the only organic carbon source in the reactors for enrichment.

### Identification of potential SAOB.

To identify potential acetate-oxidizing microorganisms, the functional genes encoding enzymes catalyzing the reversed WL pathway and the WLP-GCS pathway were searched in the annotated MAGs. Finally, the reversed WL pathway was reconstructed in seven MAGs (cz3.5, czmix2.210, czmix2.2, czmix2.30, lm1.209, lm1.58, and lmmix2.306), and the WLP-GCS pathway was reconstructed in another six MAGs (cz1.157, cz3.120, lm3.5, lm3.89, lmmix2.354, and lmmix2.72). All of these MAGs were of medium to high quality, except for MAG cz3.5 and czmix2.30, which were of low completeness (see Data Set S1).

**FIG 6:**
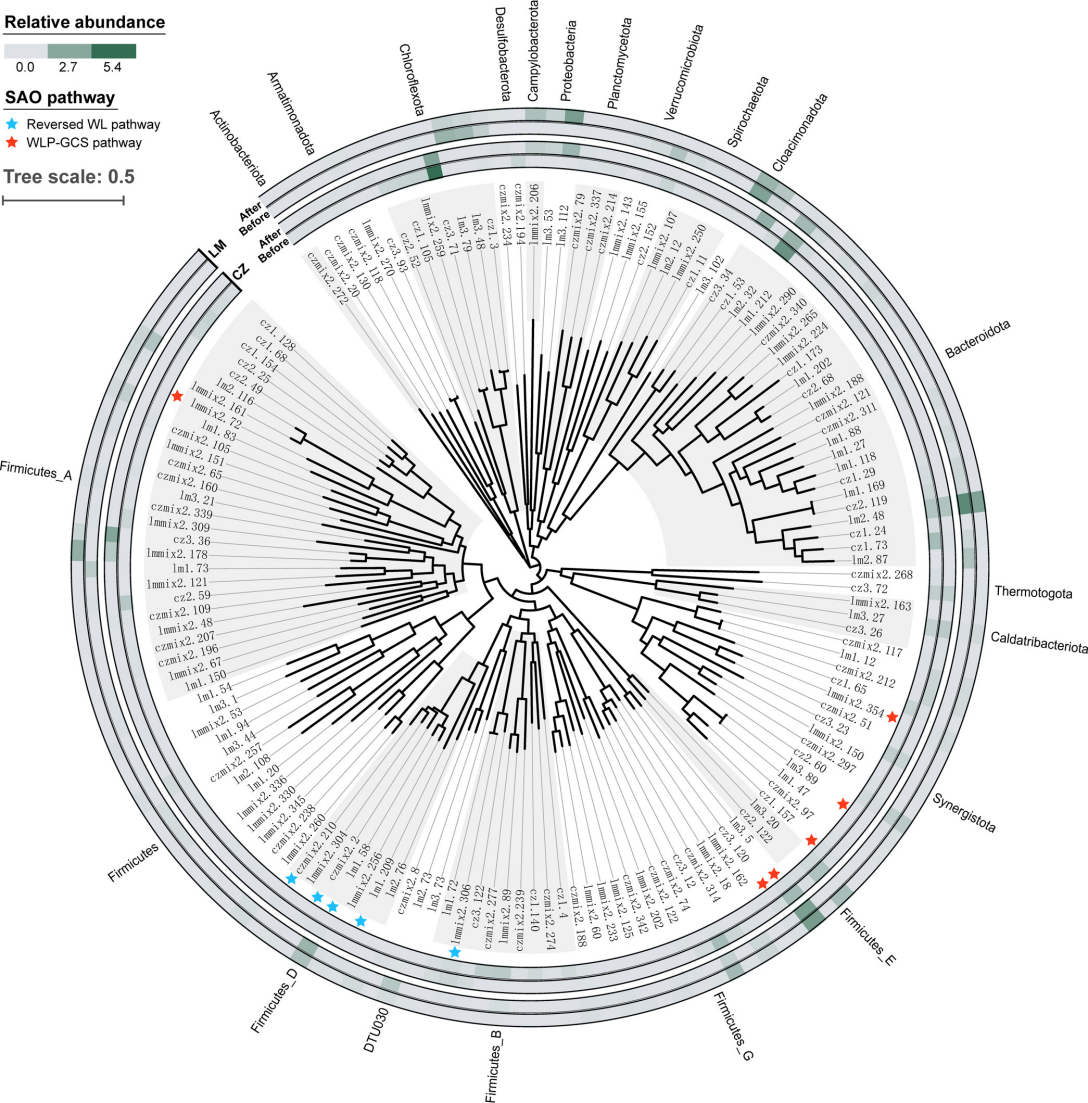
Phylogenetic tree of 146 bacterial MAGs (completeness > 80% and contamination < 5%). SAOB candidates are labeled with a star. The relative abundance of each MAG in the two digester groups, before and after the enrichment, is shown in the heatmap.

**FIG 7:**
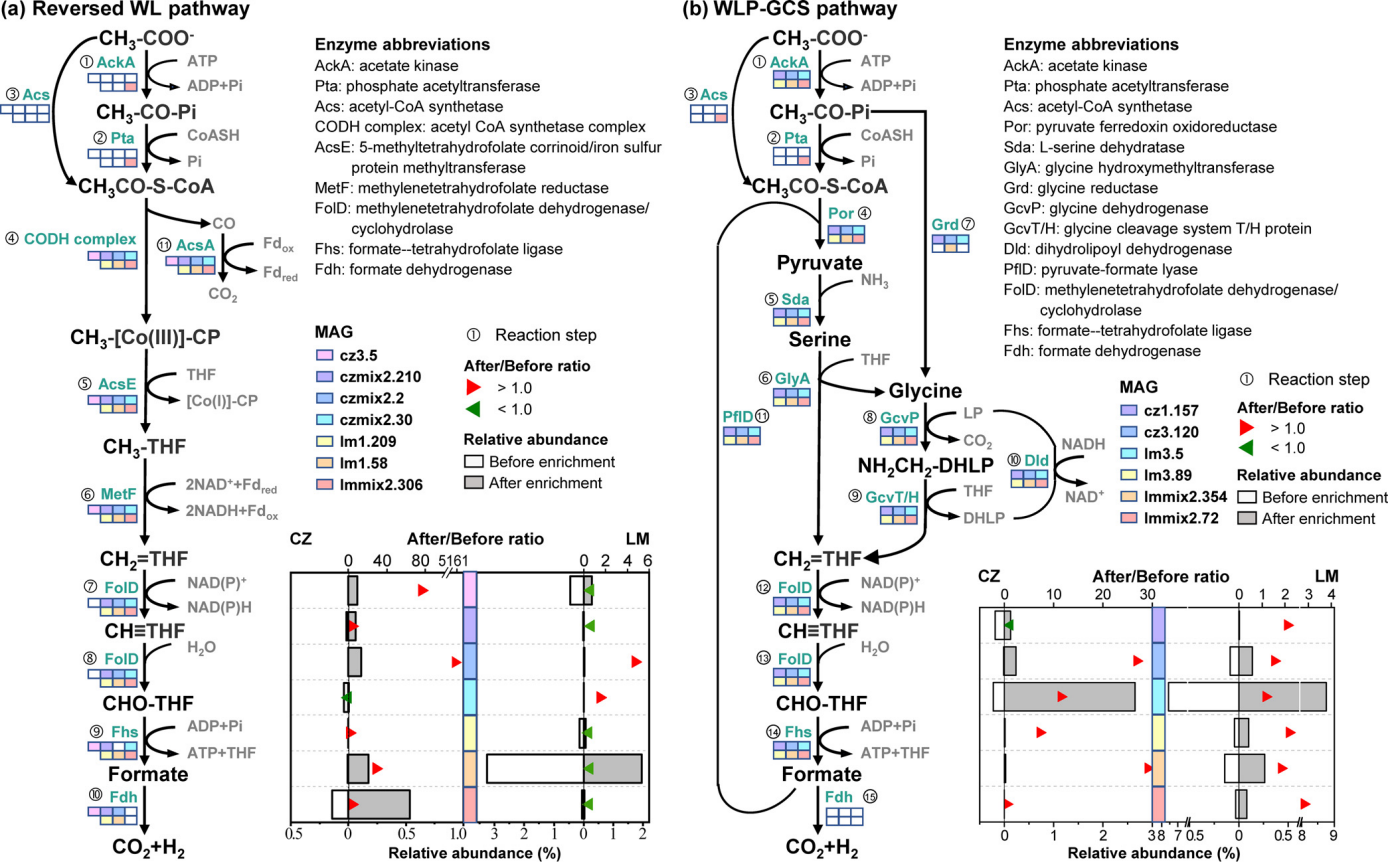
Acetate oxidation metabolism through (a) the reversed Wood-Ljungdahl (WL) pathway, and (b) the reversed Wood-Ljungdahl pathway combined with the glycine cleavage system (WLP-GCS) reconstructed from the MAGs of SAO community. The existence of related genes in these MAGs is indicated by colored rectangles. The numbers next to the genes represent the reaction steps in the pathway; details are provided in Data Set S3. The relative abundance of SAOB candidates based on metagenomic analysis (see the bottom axis) is expressed by a rectangle (open rectangles for before the enrichment and solid rectangles for after the enrichment), while the after/before ratio of the relative abundance (see the top axis) is indicated by a triangle (red triangles for >1 and green triangles for <1).

As shown in Fig. 6 and Data Set S1, the seven members performing the reversed WL pathway were all identified to be *Firmicutes*, including a known SAOB Syntrophaceticus schinkii (lmmix2.306). MAG czmix2.30, classified as DTU063 sp001512695 in the family *Tepidanaerobacteraceae*, was phylogenetically close to another known SAOB, *Tepidanaerobacter acetatoxydans* (15), and was previously proposed to be a potential SAOB (36). The other five MAGs (cz3.5, czmix2.210, czmix2.2, lm1.209, and lm1.58) were all assigned to the family DTU022, which has been reported to feature a complete or nearly complete reversed WL pathway in a previous study (36). As shown in Fig. 7a, all necessary genes directly involved in the transformation of acetyl-CoA (CH_3_CO-S-CoA) to formate could be found in MAG lmmix2.306, czmix2.30, czmix2.210, lm1.209, and lm1.58. However, the gene encoding the acetyl-CoA synthetase (Acs) that catalyzes the activation of acetate to CH_3_CO-S-CoA was missing. Alternatively, MAG lmmix2.306 showed genetic potential for a two-step activation: acetate was first transformed to acetyl phosphate (CH_3_-CO-P_i_) by acetate kinase (AckA) and then to CH_3_CO-S-CoA catalyzed by phosphate acetyltransferase (Pta).

Except for the conventional reversed WL pathway, six MAGs encoded a complete gene pool for the transformation of CH_3_CO-S-CoA to formate via the WLP-GCS pathway. All these members could activate acetate to CH_3_-CO-Pi genetically (by AckA), but only one of them (lmmix2.72) could catalyze the next step from CH_3_-CO-Pi to CH_3_CO-S-CoA (by Pta). This member also encoded the *acs* gene, which contributes to the one-step conversion. Alternatively, there was another path for CH_3_-CO-Pi to enter the glycine cleavage system, which was encoded by four of the MAGs that lacked the *pta* gene. In this pathway, CH_3_-CO-Pi was directly converted to glycine under the function of glycine reductase (Grd) (Fig. 7b), as proposed previously (14). Nevertheless, the lack of these acetate-activating genes could be because the MAGs were not 100% complete.

Among the MAGs encoding the WLP-GCS metabolism, three (MAG cz3.120, lm3.5, and lmmix2.72) belonged to the phylum *Firmicutes*, and the others (cz1.157, lm3.89, and lmmix2.354) were assigned to the phylum *Synergistota*. The *Firmicutes* members cz3.120 and lm3.5 were further classified into the uncultured genus UBA10085, and lmmix2.72 was further classified into the genus UBA1046. The function of these novel bacterial members in anaerobic digestion has seldom been described before (37). The *Synergistota* members cz1.157, lm3.89, and lmmix2.354 were assigned to the order *Synergistales*, with the first two classified to the genus *Aminivibrio*, while lmmix2.354 was assigned to uncultured *Synergistales* bacterium 58_81. Members of *Aminivibrio* have been proposed as putative SAOB in anaerobic digesters based on evidence from DNA-stable isotope probing and 16S rRNA gene amplicon sequencing (20, 38). Our results further demonstrated their SAO potential in food waste digesters. Also, this is the first time that members of *Synergistales* bacterium 58_81 have been identified as SAOB candidates.

The potential SAOB identified in this study were phylogenetically distant from those identified in thermophilic FW digesters (24, 25), indicating that temperature significantly affects the appearance of the SAO community. The results of this study showed that most of the SAOB candidates appeared in low abundance (<0.5%) before enrichment, except for lm3.5 and lm1.58, which showed high relative abundance in the LM reactor (reaching 7.6 and 3.3%, respectively). Most SAOB candidates illustrated an increase in relative abundance (based on metagenomic analysis) after enrichment compared with before, accounting for 4.01 to 11.7% of the whole communities (see Data Set S1), verifying their important roles in the cultivated systems. Specifically, after enrichment, the WLP-GCS encoding group (3.05% in the CZ reactor and 9.34% in the LM reactor) showed higher abundance than the reversed WL encoding group (0.97% in the CZ reactor and 2.35% in the LM reactor), highlighting the importance of the glycine cleavage system during acetate oxidation in anaerobic digesters.

### Microbial diversity and functional redundancy.

Starting from biomass slurry in two full-scale FW anaerobic digesters, two consortia predominated by a functional guild of syntrophic acetate oxidation together with hydrogenotrophic methanogenesis were successfully cultivated by continuous feeding with acetate under ammonia stress. The long-term enrichment simplified the microbial community and the gene pool, making acetate-related metabolisms (such as SAO and HM) become dominant, and other functions, such as syntrophic butyrate oxidation (reduced by 0.61 times in the CZ reactor and 0.94 times in the LM reactor), were less abundant. Amplicon sequencing analysis showed that *Methanoculleus* dominated the archaeal community after enrichment, while the bacterial community was still a complex mixed system due to its high metabolic functional redundancy (39). The SAO community especially demonstrated this functional redundancy. First, the conversion of acetate to CO_2_ or formate could be realized via the reversed WL pathway or the WLP-GCS pathway, as supported by metagenomic evidence. For the first step, “activation of acetate,” there could also be two paths encoded by the putative SAOB. Second, for each bacterial group with similar SAO genetic potential, several different members coexisted, which even belong to different phyla. For instance, seven members in three different families were identified to encode the reversed WL pathway, and six members in four genera have the genetic potential to perform the WLP-GCS pathway. Many factors can influence the competition between microbial members, such as acetate concentration, ammonia levels, pH, and sludge retention time (40). In our semicontinuously fed tank reactors, the slightly fluctuating acetate concentrations and gradually increasing ammonia levels might provide slightly different ecological niches for different SAOB, partially contributing to the high diversity and functional redundancy of this community. Interestingly, the SAOB candidates identified in this study showed higher abundance than the known acetate oxidizers in both reactors (see Data Set S1), which might be the true status of this functional group in engineered systems, since highly diverse SAOB candidates were reported in previous studies in different niches (13).

### Inspirations for follow-up research.

The absolute predominance of hydrogenotrophic methanogens, high value of α_C_ (>1.065), detection of hydrogen generated from acetate, and enrichment of specific functional genes all pointed to active syntrophic oxidation of acetate during the enrichment. Thirteen known or novel bacterial members, all originating from the full-scale digesters, showed genetic potential for SAO metabolism and indeed demonstrated a notable increase after enrichment. The SAOB candidates identified in this study include novel members (for instance, UBA10085 and UBA1046) that have seldom been described before (2, 13). Our previous study demonstrated that such a SAO-HM functional guild should have played an essential role in some full-scale FW digesters (such as LM), preventing failure of the system due to ammonia inhibition (3,294 to 6,810 mg/L) (23), which was considerably different from the microbiota in common sewage sludge digesters (41). In addition, strategies such as bioaugmentation with SAOB have been proven to be effective in recovering deteriorated digesters from inhibited status (42). It is therefore important to have a deep understanding of the eco-physiology of these core bacteria and methanogen members, which can help to optimize digester operation to obtain higher stability, digestion efficiency, and CH_4_ production rates. For this purpose, the *in situ* metabolic activities of the diverse SAOB, especially the energy conservation metabolism they employ, their syntrophic relationship with the partner methanogens, and their preference for different ecological niches, should be further studied. Continuous feeding, together with metagenomics and amplicon sequencing analysis, proved to be an effective way to unravel the pools of functional genes and microbial players. The genomic evidence provided a foundation for further work to study gene expression levels and activities of SAOB by using metatranscriptomics, microscopy, and other advanced methods.

### Conclusions.

Food waste digesters characterized by high ammonia levels enhanced the competitiveness of syntrophic acetate-oxidizing bacteria and hydrogenotrophic methanogens. Continuous acetate feeding under ammonia stress successfully enriched members of the SAO-HM functional guild from two separate microbiota originating from full-scale mesophilic FW digesters. While the inoculating microbiota were highly different at the beginning, during the enrichment process they gradually converged, as demonstrated by dynamic changes in the microbial community composition, the gene pool, and methane production pathways. Evidence from multiple metabolic vantages, including the absolute predominance of the hydrogenotrophic methanogen *Methanoculleus*, high value of α_C_ (>1.065), detection of generated hydrogen gas, and enrichment of involved genes, all pointed to active syntrophic oxidation of acetate during the enrichment.

The food waste anaerobic digester was proven to be a valuable pool of microbial SAOB resources, featuring high microbial diversity and rich metabolic functional redundancy. Of the 13 bacterial MAGs identified to be potential SAOB, seven can perform SAO through the conventional reversed WL pathway, which are distributed in three different bacterial families, while the other six may employ the WLP-GCS pathway, which belong to four bacterial genera. Their coexistence ensured the stable functioning of the biogas production system under ammonia stress. Specifically, the higher abundance of the WLP-GCS encoding group highlighted the importance of the glycine cleavage system during acetate oxidation in anaerobic digesters.

## MATERIALS AND METHODS

### Operation of the enrichment reactors.

Two 15-L continuously stirred tank reactors (Nalgene, USA; working volume of 10 L) were used to perform the enrichment experiment (see Fig. S6). Slurry samples obtained from two full-scale mesophilic FW anaerobic digesters (referred to as CZ and LM; see Data Set S2) were used as the inoculating microbiota. These two digesters were located at two different food waste treatment plants and demonstrated great differences in physicochemical properties (see Fig. S1) and microbial community compositions (see Fig. S4). The daily feedstock in our study was a basal medium (43) supplemented with ammonium acetate (22.0 g/L for the CZ reactor and 37.7 g/L for the LM reactor) and sodium acetate (16.7 g/L for the CZ reactor and 0 for the LM reactor) to control the organic loading rate and concentration of ammonium (NH_4_^+^) (see details in Data Set S2).

10.1128/msystems.00339-22.2 DATA SET S2 GTDB-taxo, genome information, and raw counts mapped to the middle- to high-quality MAGs, taxonomy, and relative abundances of important genera and MAGs (potential SAOB and methanogens). Download Data Set S2, XLSX file, 0.02 MB. Copyright © 2022 Li et al. 2022 Li et al. https://creativecommons.org/licenses/by/4.0/ This content is distributed under the terms of the Creative Commons Attribution 4.0 International license.

10.1128/msystems.00339-22.10 FIG S6 Schematic of the laboratory reactor (continuous stirring at 150 rpm). Download FIG S6, PDF file, 0.3 MB. Copyright © 2022 Li et al. 2022 Li et al. https://creativecommons.org/licenses/by/4.0/ This content is distributed under the terms of the Creative Commons Attribution 4.0 International license.

The laboratory reactors were operated in a semicontinuous stirring mode with a hydraulic retention time of 50 days by daily injecting 0.2 L of the modified basal medium (see Data Set S2) from the inlet using a sterile injection syringe (total volume, 60 mL) after pumping out an equivalent volume of slurry through the outlet. The slurry samples from both reactors were collected routinely and stored at −80°C for further analysis of physicochemical properties and DNA sequence, and pH was determined in a part of the liquid samples using a pH meter immediately after sampling. Gas sampling bags made up of aluminum-plastic composite film were used to collect the biogas. The temperature was controlled by a constant temperature water bath device (PC2000; Thermo Fisher Scientific, USA) at 38°C, simulating the full-scale digesters where the inoculating microbiota originated. The organic loading rate was maintained at 10 mM acetate/L/day. NH_4_^+^ in the feedstock (4,000 mg/L in CZ and 6,850 mg/L in LM) was maintained at a higher concentration than that in the full-scale digesters (1,690 mg/L in CZ and 3,960 mg/L in LM) specifically to create ammonia stress. Affected by the COVID-19 epidemic, the feeding process was interrupted twice, and the starvation period lasted for 24 and 32 days, which separated the whole enrichment process into three stages. The enrichment process lasted 228 days for the CZ reactor and 246 days for the LM reactor, including the two interruptions. During this process, slurry and gas samples were periodically taken and stored for physicochemical and biological analyses.

### Analysis of the physicochemical properties.

For the effluent samples, DAN was determined using an auto discrete analyzer (AQ2, SEAL Analytical, UK). DOC was analyzed on a total organic carbon analyzer (TOC-V CPH, Shimadzu, Japan). VFAs (including acetate, propionate, butyrate, isobutyrate, valerate, and isovalerate) were determined using a gas chromatograph (TRACE 1300; Thermo Fisher Scientific, USA) equipped with a flame ionization detector. The FAN concentration was calculated using equation 1 (44):

(1) $$\text{FAN}=(\text{TAN }\times \;10^{\text{pH}})/(\exp\left\{\frac{6,334}{273+T}\right\}+10^{\text{pH}})$$

The volume of biogas collected was measured with a gas flowmeter (SQB-0.5; Yibin Mechatronics Research Institute, China), and the composition (CH_4_, CO_2_, and H_2_) was analyzed using a gas chromatograph (Trace1300; Thermo Fisher Scientific, USA) equipped with a flame ionization detector and a thermal conductivity detector. The stable carbon isotope (^13^C) signatures of methane (δ^13^CH_4_) and carbon dioxide (δ^13^CO_2_) in biogas samples were analyzed using a gas chromatograph (Trace Ultra GC; Thermo Fisher Scientific, USA) coupled with a stable isotope ratio mass spectrometer (Delta V Advantage; Thermo Fisher Scientific, USA). α_C_ was calculated by using equation 2 (45):

(2) $$\alpha_{\text{C}}=\;(\delta^{13}{\text{CO}}_{2}+1,000)/(\delta^{13}{\text{CH}}_{4}+1,000)$$

### DNA extraction and 16S rRNA gene amplicon sequencing.

During the enrichment process, slurry samples were collected approximately every 10 days for DNA extraction and amplicon sequencing analysis. The precipitate of 0.5-mL slurry samples after centrifugation was used for total DNA extraction using the DNeasy PowerSoil kit (Qiagen, Germany) according to the manufacturer’s instructions. The V4 region of the 16S rRNA gene was amplified with the primer pair 515F/806R (46) using an ABI GeneAmp 9700 PCR thermocycler (ABI, CA, USA) and TransStart Fastpfu DNA polymerase. The PCR program consisted of an initial denaturation at 95°C for 3 min; followed by 27 cycles of denaturation (95°C for 30 s), annealing (55°C for 30 s), and extension (72°C for 45 s); and then a final extension at 72°C for 10 min. The PCR product was extracted from a 2% agarose gel and purified using the AxyPrep DNA gel extraction kit (Axygen Biosciences, Union City, CA) according to the manufacturer’s instructions and quantified by using a Quantus fluorometer (Promega, USA). Purified amplicons were pooled in equimolar amounts and paired-end sequenced (2 × 300 bp) on an Illumina MiSeq PE300 platform (Illumina, San Diego, CA) according to the standard protocols. Amplification, library preparation, and sequencing were performed by Majorbio Bio-pharm Technology Co., Ltd., Shanghai, China. Raw sequence data files were analyzed within the QIIME2 (2020.2) system (47). Trimming, denoising, and merging of the sequences were performed using the q2-dada2 plugin (48), and an amplicon sequence variant table with 30,033 to 56,741 assembled sequences per sample was obtained. Taxonomic assignments of the ASVs were then carried out by using classify-sklearn in the q2-feature-classifier plugin according to the SILVA database release 138 (49).

### Metagenomic sequencing.

Metagenomic sequencing was conducted using six slurry samples, which were taken 2 months before the enrichment experiment (CZ1 and LM1 were taken from the full-scale digesters), simultaneously with the start of the experiment (CZ2 and LM2 taken from the full-scale digesters), and at the end of the experimental period (CZ3 and LM3 taken from the lab-scale reactors). Purified DNA extracts were used to prepare sequencing libraries using the NEBNext Ultra DNA Library Prep kit for Illumina (New England Biolabs, USA). The libraries were paired-end (2 × 150 bp) sequenced on the Illumina NovaSeq 6000 platform using the HiSeq Rapid PE cluster kit v2 and the HiSeq Rapid SBS kit v2 (500 cycles) in rapid run mode. Metagenomic sequencing was performed by Novogene Co., Ltd., Beijing, China. Details of the experimental procedure of metagenomic sequencing can be found in Text S1.

10.1128/msystems.00339-22.4 TEXT S1 Details of the experimental procedure of metagenomic sequencing. Download Text S1, DOCX file, 0.03 MB. Copyright © 2022 Li et al. 2022 Li et al. https://creativecommons.org/licenses/by/4.0/ This content is distributed under the terms of the Creative Commons Attribution 4.0 International license.

### Metagenomic analysis.

Raw metagenomic reads were first processed using fastp to remove the barcodes and adapters and further trimmed using the READ_QC module of MetaWRAP pipeline v.1.2.1 (50). The trimmed reads were assembled into six single assemblies and two coassemblies with the ASSEMBLY module in MetaWRAP. Further binning of the contigs in each assembly was performed with a hybrid approach offered by MetaWRAP using metaBAT2 (51), CONCOCT (52), and MaxBin2 (53) in the BINNING module. Bins generated with all three methods were refined, and the representative ones were retained within the bin_refinement plugin, resulting in eight sets of metagenomic bins (see Data Set S2). The completeness and contamination of each bin were assessed using CheckM v1.1.3 (54). All these bins were then dereplicated with dRep v.3.2.0 (55) to generate 1,534 unique MAGs. Objective taxonomic classifications were assigned to the MAGs according to the Genome Taxonomy DataBase (GTDB) taxonomy (release R95) using the toolkit GTDB-Tk v1.4.1 (56).

### Gene prediction.

The MAGs were annotated using Prokka v1.14.6 (57) with the Bacteria or Archaea database based on their taxonomic classification (58). An E value threshold of 10^−6^ was used for the prediction of open reading frames.

### Quantification of MAGs and genes.

The relative abundance of each MAG or open reading frame was estimated by mapping the trimmed metagenomic reads to the contigs of each MAG or the open reading frame using the CLC genomics workbench (version 21.05), using the Map Reads to Reference function with length fraction = 0.75, and similarity fraction = 0.95. The coverage values were calculated by considering the total length of the MAGs or open reading frames.

### Pathway analysis.

The entire open reading frame set of the MAGs was further analyzed using EnrichM v0.6.3 and annotated with KEGG numbers for further metabolic pathway analysis (59). Among them, those involved in the degradation of acetate via the reversed WL pathway (60) or WLP-GCS pathway (9) and the production of methane via the AM, HM, and MM pathways were selected based on a list of gene families (determined by KO) that are directly associated with the corresponding KEGG metabolisms (see Data Set S3).

### Phylogenetic tree of the genomes.

Only the bacterial and archaeal MAGs with medium to high quality (completeness > 80%, contamination < 5%) were selected to construct the phylogenetic genome trees, which were created using GTDB-Tk (56). iTOL (61) was used for further tree refinements.

### Identification of important genes or MAGs.

To identify the genes or MAGs that were significantly enriched during the process, the ratio of the relative abundance of genes or MAGs after and before the enrichment process (defined as the “after/before ratio” in Fig. 5 and 7; see also Data Set S3) was calculated. In this way, genes or MAGs with after/before ratios of >2.0 were identified as enriched genes, while those with ratios of <0.5 were considered degenerated genes.

### Data availability.

All sequencing data have been submitted to the National Microbiology Data Center (http://nmdc.cn/) under the BioProject ID NMDC10017957 (see details in Data Set S2), which contains raw sequences of six metagenomes (NMDC Accession NMDC40014603 to NMDC40014608) and twenty-four 16S rRNA gene amplicon data sets (NMDC Accession NMDC40024651 to NMDC40024674).

## References

[1] Mountfort DO, Asher RA. 1978. Changes in proportions of acetate and carbon dioxide used as methane precursors during the anaerobic digestion of bovine waste. Appl Environ Microbiol 35:648–654. doi:10.1128/aem.35.4.648-654.1978. 565615 PMC242900

[2] Dyksma S, Jansen L, Gallert C. 2020. Syntrophic acetate oxidation replaces acetoclastic methanogenesis during thermophilic digestion of biowaste. Microbiome 8:105. doi:10.1186/s40168-020-00862-5. 32620171 PMC7334858

[3] Hattori S. 2008. Syntrophic acetate-oxidizing microbes in methanogenic environments. Microbes Environ 23:118–127. doi:10.1264/jsme2.23.118. 21558697

[4] Fuchs W, Wang XM, Gabauer W, Ortner M, Li ZF. 2018. Tackling ammonia inhibition for efficient biogas production from chicken manure: status and technical trends in Europe and China. Renewable & Sustainable Energy Rev 97:186–199. doi:10.1016/j.rser.2018.08.038.

[5] Bi S, Westerholm M, Hu W, Mahdy A, Dong T, Sun Y, Qiao W, Dong R. 2021. The metabolic performance and microbial communities of anaerobic digestion of chicken manure under stressed ammonia condition: a case study of a 10-year successful biogas plant. Renewable Energy 167:644–651. doi:10.1016/j.renene.2020.11.133.

[6] Lü F, Hao L, Guan D, Qi Y, Shao L, He P. 2013. Synergetic stress of acids and ammonium on the shift in the methanogenic pathways during thermophilic anaerobic digestion of organics. Water Res 47:2297–2306. doi:10.1016/j.watres.2013.01.049. 23434042

[7] Yan M, Treu L, Zhu X, Tian H, Basile A, Fotidis IA, Campanaro S, Angelidaki I. 2020. Insights into ammonia adaptation and methanogenic precursor oxidation by genome-centric analysis. Environ Sci Technol 54:12568–12582. doi:10.1021/acs.est.0c01945. 32852203 PMC8154354

[8] Westerholm M, Dolfing J, Schnurer A. 2019. Growth characteristics and thermodynamics of syntrophic acetate oxidizers. Environ Sci Technol 53:5512–5520. doi:10.1021/acs.est.9b00288. 30990997

[9] Nobu MK, Narihiro T, Rinke C, Kamagata Y, Tringe SG, Woyke T, Liu W-T. 2015. Microbial dark matter ecogenomics reveals complex synergistic networks in a methanogenic bioreactor. ISME J 9:1710–1722. doi:10.1038/ismej.2014.256. 25615435 PMC4511927

[10] Westerholm M, Roos S, Schnürer A. 2010. *Syntrophaceticus schinkii* gen. nov., sp. nov., an anaerobic, syntrophic acetate-oxidizing bacterium isolated from a mesophilic anaerobic filter. FEMS Microbiol Lett 309:100–104. doi:10.1111/j.1574-6968.2010.02023.x. 20546311

[11] Westerholm M, Roos S, Schnürer A. 2011. *Tepidanaerobacter acetatoxydans* sp. nov., an anaerobic, syntrophic acetate-oxidizing bacterium isolated from two ammonium-enriched mesophilic methanogenic processes. Syst Appl Microbiol 34:260–266. doi:10.1016/j.syapm.2010.11.018. 21498020

[12] Hattori S, Kamagata Y, Hanada S, Shoun H. 2000. *Thermacetogenium phaeum* gen. nov., sp. nov., a strictly anaerobic, thermophilic, syntrophic acetate-oxidizing bacterium. Int J Syst Evol Microbiol 50:1601–1609. doi:10.1099/00207713-50-4-1601. 10939667

[13] Westerholm M, Moestedt J, Schnürer A. 2016. Biogas production through syntrophic acetate oxidation and deliberate operating strategies for improved digester performance. Applied Energy 179:124–135. doi:10.1016/j.apenergy.2016.06.061.

[14] Zhu X, Campanaro S, Treu L, Seshadri R, Ivanova N, Kougias PG, Kyrpides N, Angelidaki I. 2020. Metabolic dependencies govern microbial syntrophies during methanogenesis in an anaerobic digestion ecosystem. Microbiome 8:22–22. doi:10.1186/s40168-019-0780-9. 32061251 PMC7024554

[15] Campanaro S, Treu L, Kougias PG, Luo G, Angelidaki I. 2018. Metagenomic binning reveals the functional roles of core abundant microorganisms in twelve full-scale biogas plants. Water Res 140:123–134. doi:10.1016/j.watres.2018.04.043. 29704757

[16] Ito T, Yoshiguchi K, Ariesyady HD, Okabe S. 2011. Identification of a novel acetate-utilizing bacterium belonging to *Synergistes* group 4 in anaerobic digester sludge. ISME J 5:1844–1856. doi:10.1038/ismej.2011.59. 21562600 PMC3223300

[17] Mosbæk F, Kjeldal H, Mulat DG, Albertsen M, Ward AJ, Feilberg A, Nielsen JL. 2016. Identification of syntrophic acetate-oxidizing bacteria in anaerobic digesters by combined protein-based stable isotope probing and metagenomics. ISME J 10:2405–2418. doi:10.1038/ismej.2016.39. 27128991 PMC5030692

[18] Frank JA, Arntzen MØ, Sun L, Hagen LH, McHardy AC, Horn SJ, Eijsink VGH, Schnürer A, Pope PB. 2016. Novel syntrophic populations dominate an ammonia-tolerant methanogenic microbiome. mSystems 1:e00092-16. doi:10.1128/mSystems.00092-16. 27822555 PMC5080403

[19] Westerholm M, Müller B, Singh A, Karlsson Lindsjö O, Schnürer A. 2018. Detection of novel syntrophic acetate-oxidizing bacteria from biogas processes by continuous acetate enrichment approaches. Microb Biotechnol 11:680–693. doi:10.1111/1751-7915.13035. 29239113 PMC6011928

[20] Yi Y, Wang H, Chen Y, Gou M, Xia Z, Hu B, Nie Y, Tang Y. 2020. Identification of novel butyrate- and acetate-oxidizing bacteria in butyrate-fed mesophilic anaerobic chemostats by DNA-based stable isotope probing. Microb Ecol 79:285–298. doi:10.1007/s00248-019-01400-z. 31263981

[21] Ho D, Jensen P, Batstone D. 2014. Effects of temperature and hydraulic retention time on acetotrophic pathways and performance in high-rate sludge digestion. Environ Sci Technol 48:6468–6476. doi:10.1021/es500074j. 24797677

[22] Han WH, He PJ, Lin YC, Shao LM, Lu F. 2019. A methanogenic consortium was active and exhibited long-term survival in an extremely acidified thermophilic bioreactor. Front Microbiol 10:15. doi:10.3389/fmicb.2019.02757. 32038509 PMC6988822

[23] Li C, He P, Hao L, Lü F, Shao L, Zhang H. 2022. Diverse acetate-oxidizing syntrophs contributing to biogas production from food waste in full-scale anaerobic digesters in China. Renewable Energy 193:240–250. doi:10.1016/j.renene.2022.04.143.

[24] Zheng D, Wang H, Gou M, Nobu MK, Narihiro T, Hu B, Nie Y, Tang Y. 2019. Identification of novel potential acetate-oxidizing bacteria in thermophilic methanogenic chemostats by DNA stable isotope probing. Appl Microbiol Biotechnol 103:8631–8645. doi:10.1007/s00253-019-10078-9. 31418053

[25] Zeng Y, Zheng D, Gou M, Xia Z, Chen Y, Nobu MK, Tang Y. 2021. Chasing the metabolism of novel syntrophic acetate-oxidizing bacteria in thermophilic methanogenic chemostats. bioRxiv. https://www.biorxiv.org/content/10.1101/2021.07.06.451242v1. 10.1128/aem.01090-23 PMC10880629 38259075

[26] Singh A, Schnürer A, Westerholm M. 2021. Enrichment and description of novel bacteria performing syntrophic propionate oxidation at high ammonia level. Environ Microbiol 23:1620–1637. doi:10.1111/1462-2920.15388. 33400377

[27] Hao L, Michaelsen TY, Singleton CM, Dottorini G, Kirkegaard RH, Albertsen M, Nielsen PH, Dueholm MS. 2020. Novel syntrophic bacteria in full-scale anaerobic digesters revealed by genome-centric metatranscriptomics. ISME J 14:906–918. doi:10.1038/s41396-019-0571-0. 31896784 PMC7082340

[28] de Jonge N, Moset V, Møller HB, Nielsen JL. 2017. Microbial population dynamics in continuous anaerobic digester systems during start up, stable conditions and recovery after starvation. Bioresour Technol 232:313–320. doi:10.1016/j.biortech.2017.02.036. 28242388

[29] Peces M, Astals S, Jensen PD, Clarke WP. 2018. Deterministic mechanisms define the long-term anaerobic digestion microbiome and its functionality regardless of the initial microbial community. Water Res 141:366–376. doi:10.1016/j.watres.2018.05.028. 29807319

[30] Hubert C, Voordouw G. 2007. Oil field souring control by nitrate-reducing *Sulfurospirillum* spp. that outcompete sulfate-reducing bacteria for organic electron donors. Appl Environ Microbiol 73:2644–2652. doi:10.1128/AEM.02332-06. 17308184 PMC1855586

[31] Conrad R. 2005. Quantification of methanogenic pathways using stable carbon isotopic signatures: a review and a proposal. Organic Geochem 36:739–752. doi:10.1016/j.orggeochem.2004.09.006.

[32] Fey A, Claus P, Conrad R. 2004. Temporal change of ^13^C-isotope signatures and methanogenic pathways in rice field soil incubated anoxically at different temperatures. Geochim Cosmochim Acta 68:293–306. doi:10.1016/S0016-7037(03)00426-5.

[33] Rotaru A-E, Shrestha PM, Liu F, Shrestha M, Shrestha D, Embree M, Zengler K, Wardman C, Nevin KP, Lovley DR. 2014. A new model for electron flow during anaerobic digestion: direct interspecies electron transfer to *Methanosaeta* for the reduction of carbon dioxide to methane. Energy Environ Sci 7:408–415. doi:10.1039/C3EE42189A.

[34] Hao L, Fan L, Chapleur O, Guenne A, Bize A, Bureau C, Lü F, He P, Bouchez T, Mazéas L. 2021. Gradual development of ammonia-induced syntrophic acetate-oxidizing activities under mesophilic and thermophilic conditions quantitatively tracked using multiple isotopic approaches. Water Res 204:117586. doi:10.1016/j.watres.2021.117586. 34474248

[35] Borrel G, Parisot N, Harris HMB, Peyretaillade E, Gaci N, Tottey W, Bardot O, Raymann K, Gribaldo S, Peyret P, O’Toole PW, Brugère J-F. 2014. Comparative genomics highlights the unique biology of *Methanomassiliicoccales*, a *Thermoplasmatales*-related seventh order of methanogenic archaea that encodes pyrrolysine. BMC Genomics 15:679. doi:10.1186/1471-2164-15-679. 25124552 PMC4153887

[36] Campanaro S, Treu L, Kougias PG, De Francisci D, Valle G, Angelidaki I. 2016. Metagenomic analysis and functional characterization of the biogas microbiome using high throughput shotgun sequencing and a novel binning strategy. Biotechnol Biofuels 9:26. doi:10.1186/s13068-016-0441-1. 26839589 PMC4736482

[37] Campanaro S, Treu L, Rodriguez-R LM, Kovalovszki A, Ziels RM, Maus I, Zhu X, Kougias PG, Basile A, Luo G, Schlüter A, Konstantinidis KT, Angelidaki I. 2020. New insights from the biogas microbiome by comprehensive genome-resolved metagenomics of nearly 1600 species originating from multiple anaerobic digesters. Biotechnol Biofuels 13:25. doi:10.1186/s13068-020-01679-y. 32123542 PMC7038595

[38] Aoyagi T, Inaba T, Aizawa H, Mayumi D, Sakata S, Charfi A, Suh C, Lee JH, Sato Y, Ogata A, Habe H, Hori T. 2020. Unexpected diversity of acetate degraders in anaerobic membrane bioreactor treating organic solid waste revealed by high-sensitivity stable isotope probing. Water Res 176:115750. doi:10.1016/j.watres.2020.115750. 32272322

[39] Louca S, Polz MF, Mazel F, Albright MBN, Huber JA, O’Connor MI, Ackermann M, Hahn AS, Srivastava DS, Crowe SA, Doebeli M, Parfrey LW. 2018. Function and functional redundancy in microbial systems. Nat Ecol Evol 2:936–943. doi:10.1038/s41559-018-0519-1. 29662222

[40] Ju F, Lau F, Zhang T. 2017. Linking microbial community, environmental variables, and methanogenesis in anaerobic biogas digesters of chemically enhanced primary treatment sludge. Environ Sci Technol 51:3982–3992. doi:10.1021/acs.est.6b06344. 28240534

[41] Hao L, Bize A, Conteau D, Chapleur O, Courtois S, Kroff P, Desmond-Le Quemener E, Bouchez T, Mazeas L. 2016. New insights into the key microbial phylotypes of anaerobic sludge digesters under different operational conditions. Water Res 102:158–169. doi:10.1016/j.watres.2016.06.014. 27340817

[42] Yang Z, Wang W, Liu C, Zhang R, Liu G. 2019. Mitigation of ammonia inhibition through bioaugmentation with different microorganisms during anaerobic digestion: selection of strains and reactor performance evaluation. Water Res 155:214–224. doi:10.1016/j.watres.2019.02.048. 30849735

[43] Lin Y, Lü F, Shao L, He P. 2013. Influence of bicarbonate buffer on the methanogenetic pathway during thermophilic anaerobic digestion. Bioresour Technol 137:245–253. doi:10.1016/j.biortech.2013.03.093. 23587826

[44] Anthonisen AC, Loehr RC, Prakasam TBS, Srinath EG. 1976. Inhibition of nitrification by ammonia and nitrous acid. J Water Pollut Control Fed 48:835–852. 948105

[45] Whiticar MJ, Faber E, Schoell M. 1986. Biogenic methane formation in marine and freshwater environments: CO_2_ reduction versus acetate fermentation—isotope evidence. Geochim Cosmochim Acta 50:693–709. doi:10.1016/0016-7037(86)90346-7.

[46] Caporaso JG, Lauber CL, Walters WA, Berg-Lyons D, Huntley J, Fierer N, Owens SM, Betley J, Fraser L, Bauer M, Gormley N, Gilbert JA, Smith G, Knight R. 2012. Ultra-high-throughput microbial community analysis on the Illumina HiSeq and MiSeq platforms. ISME J 6:1621–1624. doi:10.1038/ismej.2012.8. 22402401 PMC3400413

[47] Bolyen E, Rideout JR, Dillon MR, Bokulich NA, Abnet CC, Al-Ghalith GA, Alexander H, Alm EJ, Arumugam M, Asnicar F, Bai Y, Bisanz JE, Bittinger K, Brejnrod A, Brislawn CJ, Brown CT, Callahan BJ, Caraballo-Rodríguez AM, Chase J, Cope EK, Da Silva R, Diener C, Dorrestein PC, et al. 2019. Reproducible, interactive, scalable and extensible microbiome data science using QIIME 2. Nat Biotechnol 37:852–857. doi:10.1038/s41587-019-0209-9. 31341288 PMC7015180

[48] Callahan BJ, McMurdie PJ, Rosen MJ, Han AW, Johnson AJA, Holmes SP. 2016. DADA2: high-resolution sample inference from Illumina amplicon data. Nat Methods 13:581–583. doi:10.1038/nmeth.3869. 27214047 PMC4927377

[49] Quast C, Pruesse E, Yilmaz P, Gerken J, Schweer T, Yarza P, Peplies J, Glöckner FO. 2013. The SILVA ribosomal RNA gene database project: improved data processing and web-based tools. Nucleic Acids Res 41:D590–D596. doi:10.1093/nar/gks1219. 23193283 PMC3531112

[50] Uritskiy GV, DiRuggiero J, Taylor J. 2018. MetaWRAP: a flexible pipeline for genome-resolved metagenomic data analysis. Microbiome 6:1–13. doi:10.1186/s40168-018-0541-1. 30219103 PMC6138922

[51] Kang DD, Li F, Kirton E, Thomas A, Egan R, An H, Wang Z. 2019. MetaBAT 2: an adaptive binning algorithm for robust and efficient genome reconstruction from metagenome assemblies. PeerJ 7:e7359. doi:10.7717/peerj.7359. 31388474 PMC6662567

[52] Alneberg J, Bjarnason BS, de Bruijn I, Schirmer M, Quick J, Ijaz UZ, Lahti L, Loman NJ, Andersson AF, Quince C. 2014. Binning metagenomic contigs by coverage and composition. Nat Methods 11:1144–1146. doi:10.1038/nmeth.3103. 25218180

[53] Wu Y-W, Simmons BA, Singer SW. 2016. MaxBin 2.0: an automated binning algorithm to recover genomes from multiple metagenomic datasets. Bioinformatics 32:605–607. doi:10.1093/bioinformatics/btv638. 26515820

[54] Parks DH, Imelfort M, Skennerton CT, Hugenholtz P, Tyson GW. 2015. CheckM: assessing the quality of microbial genomes recovered from isolates, single cells, and metagenomes. Genome Res 25:1043–1055. doi:10.1101/gr.186072.114. 25977477 PMC4484387

[55] Olm MR, Brown CT, Brooks B, Banfield JF. 2017. dRep: a tool for fast and accurate genomic comparisons that enables improved genome recovery from metagenomes through de-replication. ISME J 11:2864–2868. doi:10.1038/ismej.2017.126. 28742071 PMC5702732

[56] Chaumeil P-A, Mussig AJ, Hugenholtz P, Parks DH. 2020. GTDB-Tk: a toolkit to classify genomes with the Genome Taxonomy Database. Oxford University Press, Oxford, United Kingdom. 10.1093/bioinformatics/btz848 PMC7703759 31730192

[57] Seemann T. 2014. Prokka: rapid prokaryotic genome annotation. Bioinformatics 30:2068–2069. doi:10.1093/bioinformatics/btu153. 24642063

[58] Parks DH, Chuvochina M, Waite DW, Rinke C, Skarshewski A, Chaumeil P-A, Hugenholtz P. 2018. A standardized bacterial taxonomy based on genome phylogeny substantially revises the tree of life. Nat Biotechnol 36:996–1004. doi:10.1038/nbt.4229. 30148503

[59] Kanehisa M, Goto S, Sato Y, Kawashima M, Furumichi M, Tanabe M. 2014. Data, information, knowledge and principle: back to metabolism in KEGG. Nucleic Acids Res 42:D199–D205. doi:10.1093/nar/gkt1076. 24214961 PMC3965122

[60] Müller B, Sun L, Schnürer A. 2013. First insights into the syntrophic acetate-oxidizing bacteria – a genetic study. Microbiologyopen 2:35–53. doi:10.1002/mbo3.50. 23239474 PMC3584212

[61] Letunic I, Bork P. 2021. Interactive Tree Of Life (iTOL) v5: an online tool for phylogenetic tree display and annotation. Nucleic Acids Res 49:W293–W296. doi:10.1093/nar/gkab301. 33885785 PMC8265157

