# Supplementary material for: Syntrophic Acetate-Oxidizing Microbial Consortia Enriched from Full-Scale Mesophilic Food Waste Anaerobic Digesters Showing High Biodiversity and Functional Redundancy
Source: mSystems. 2022 Sep 8;7(5):e00339-22. doi: 10.1128/msystems.00339-22 (PMC9600251; doi:10.1128/msystems.00339-22)
Supplement: FIG S1 [file msystems.00339-22-s0001.pdf]

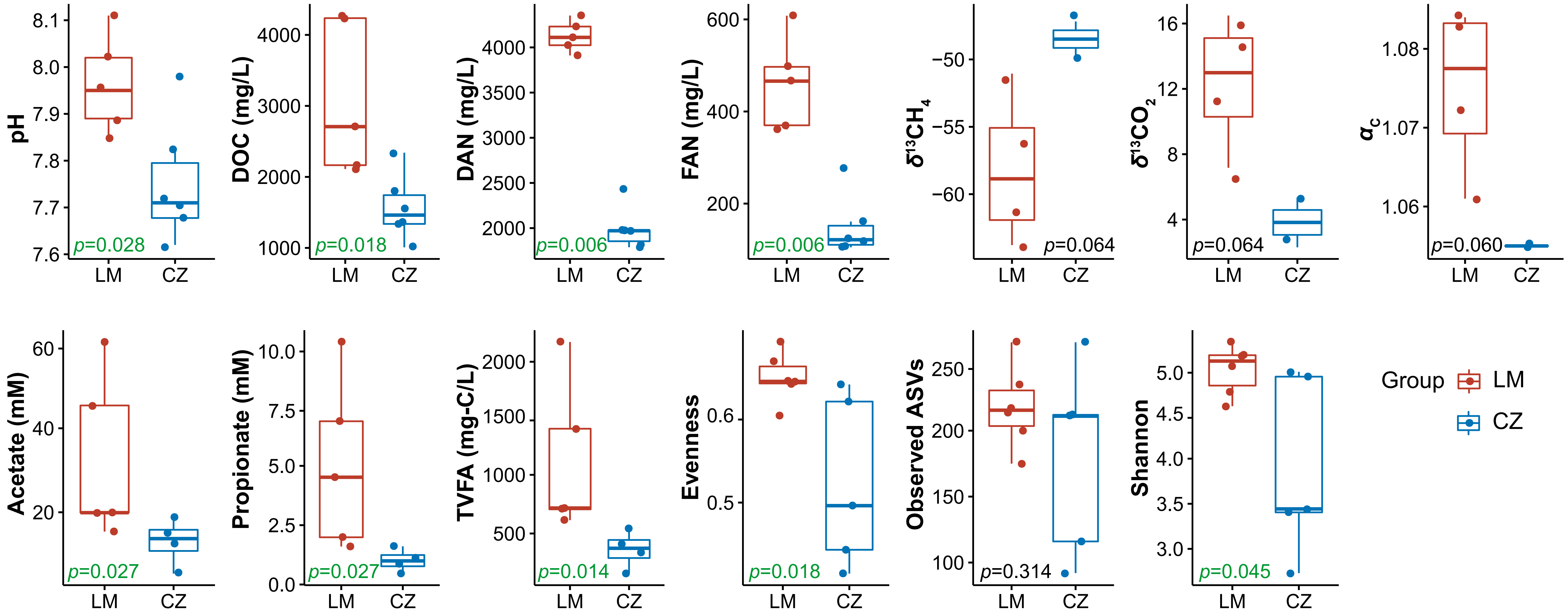

Figure S1. Comparison of physicochemical properties and microbial diversities (based on 16S rRNA gene amplicon data) of full-scale anaerobic digesters where the inoculating microbiota come from. Kruskal-Wallis test was performed using the FSA package in R to verify the statistical significance.
