# Supplementary material for: Syntrophic Acetate-Oxidizing Microbial Consortia Enriched from Full-Scale Mesophilic Food Waste Anaerobic Digesters Showing High Biodiversity and Functional Redundancy
Source: mSystems. 2022 Sep 8;7(5):e00339-22. doi: 10.1128/msystems.00339-22 (PMC9600251; doi:10.1128/msystems.00339-22)
Supplement: FIG S2 [file msystems.00339-22-s0002.pdf]

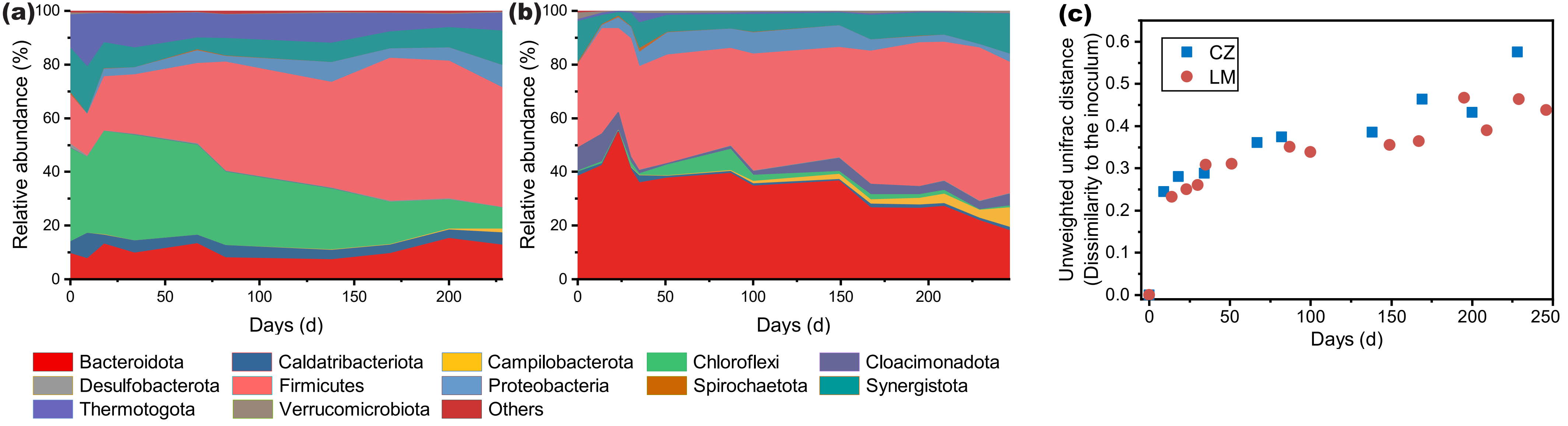

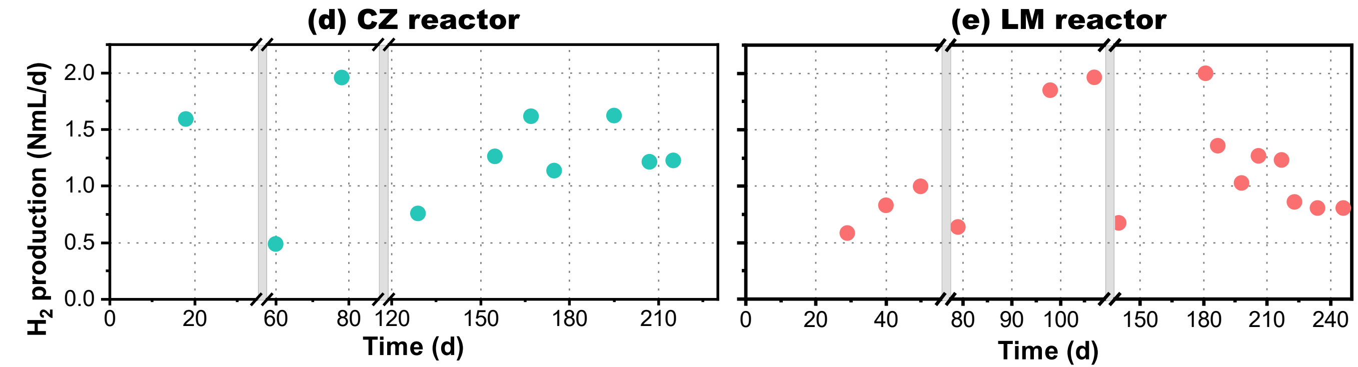

Figure S2. Area chart representing the relative abundance of the top 12 bacterial phyla (based on 16S rRNA gene amplicon data) in (a) CZ reactor, and (b) LM reactor during the enrichment process. Only phyla with any occurrence > 1% in at least one sample are shown. (c) Dynamic change of compositional dissimilarity of the microbial communities (based on 16S rRNA gene amplicon data) in CZ reactor and LM reactor. Expressed as unweighted Unifrac distance relative to the inoculum. Amount of hydrogen in the collected biogas during the enrichment process in CZ reactor (d) and LM reactor (e).
