# Supplementary material for: Syntrophic Acetate-Oxidizing Microbial Consortia Enriched from Full-Scale Mesophilic Food Waste Anaerobic Digesters Showing High Biodiversity and Functional Redundancy
Source: mSystems. 2022 Sep 8;7(5):e00339-22. doi: 10.1128/msystems.00339-22 (PMC9600251; doi:10.1128/msystems.00339-22)
Supplement: FIG S3 [file msystems.00339-22-s0003.pdf]

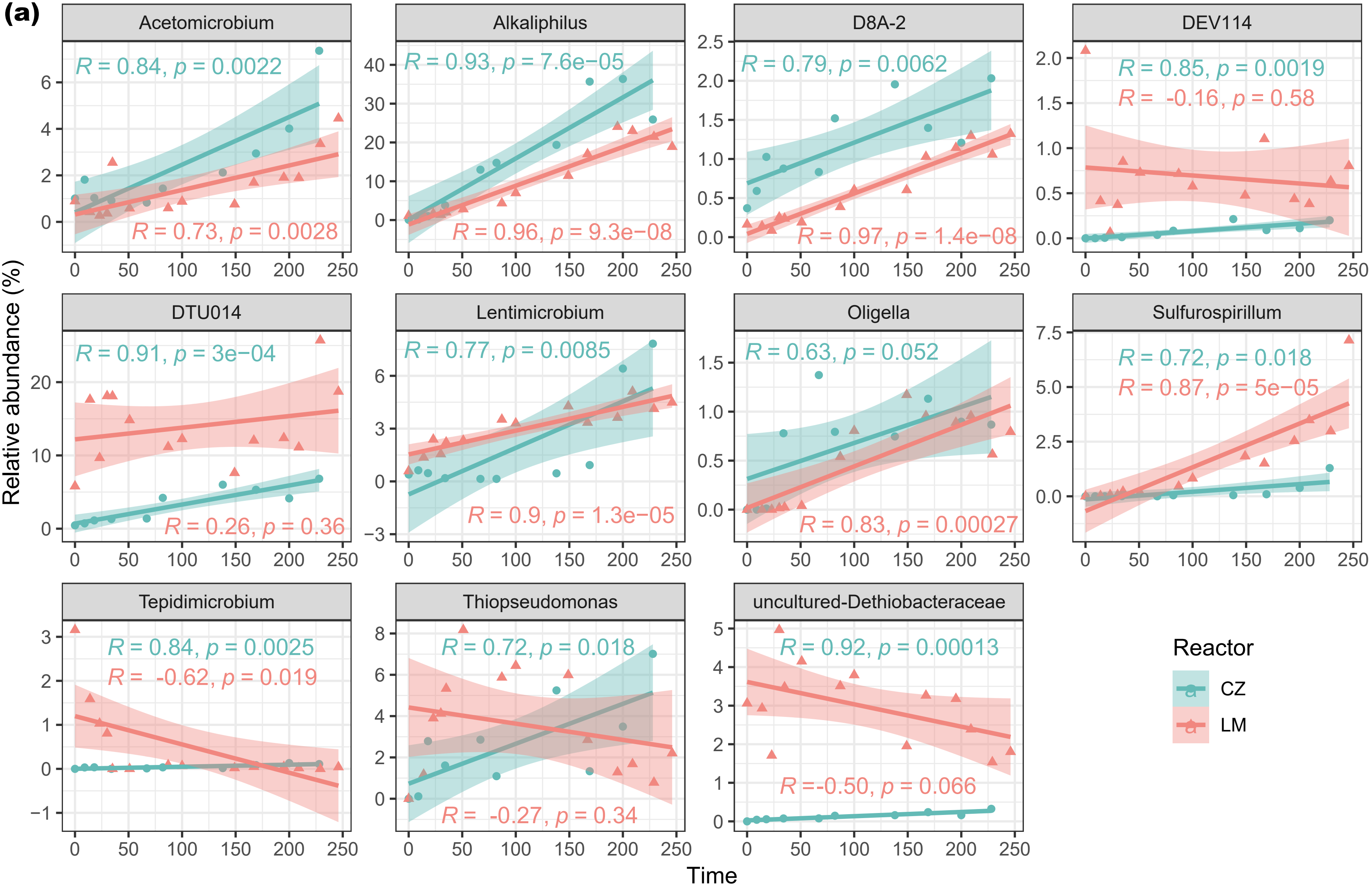

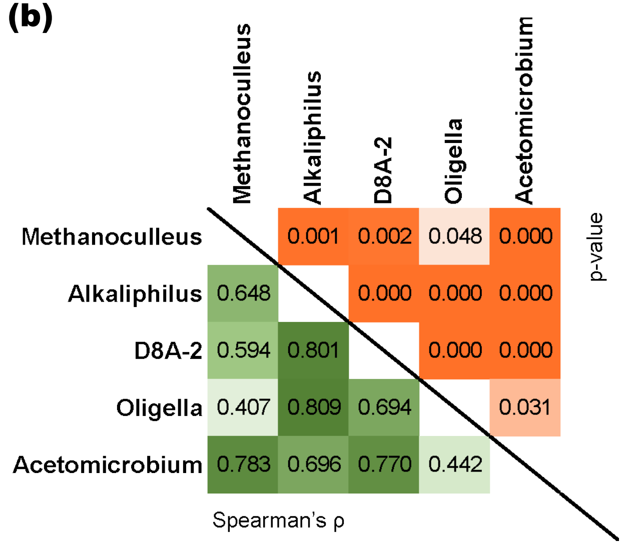

Figure S3. (a) Dynamic changes of relative abundance of selected bacterial genera in the two lab-scale reactors. 11 abundant genera (with relative abundance > 1% of the total bacterial sequences in at least one sample based on 16S rRNA gene amplicon data) which significantly increased in relative abundance over enrichment time in at least one reactor are shown. Spearman's correlation coefficient and significance evaluated by two-tailed test are given. (b) Correlations between the Methanoculleus and bacterial members Alkaliphilus, D8A-2, Oligella, and Acetomicrobium based on amplicon data analysis.
