# Supplementary material for: Syntrophic Acetate-Oxidizing Microbial Consortia Enriched from Full-Scale Mesophilic Food Waste Anaerobic Digesters Showing High Biodiversity and Functional Redundancy
Source: mSystems. 2022 Sep 8;7(5):e00339-22. doi: 10.1128/msystems.00339-22 (PMC9600251; doi:10.1128/msystems.00339-22)
Supplement: FIG S4 [file msystems.00339-22-s0004.pdf]

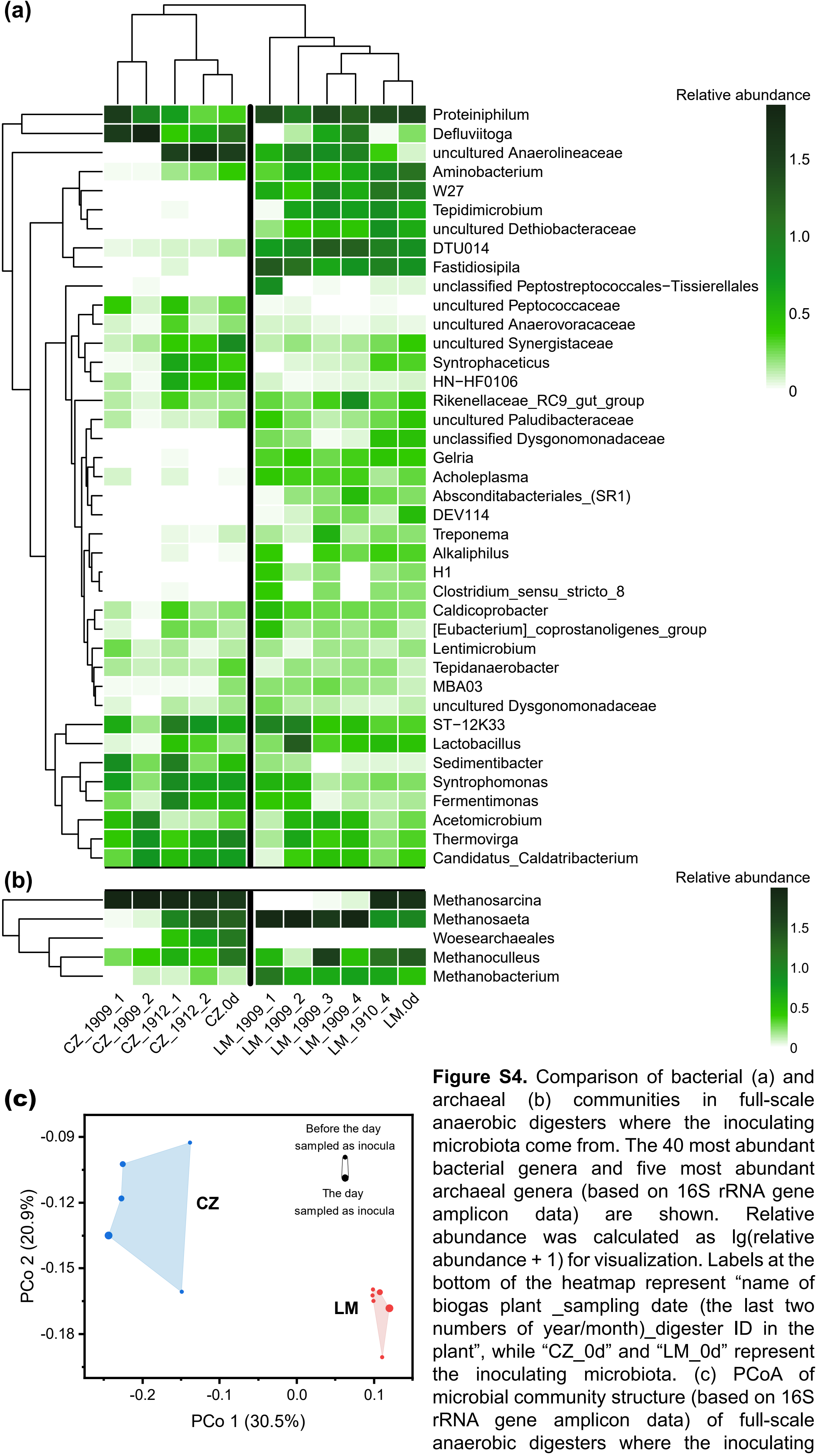

Figure S4. Comparison of bacterial (a) and archaeal (b) communities in full-scale anaerobic digesters where the inoculating microbiota come from. The 40 most abundant bacterial genera and five most abundant archaeal genera (based on 16S rRNA gene amplicon data) are shown. Relative abundance was calculated as lg(relative abundance + 1) for visualization. Labels at the bottom of the heatmap represent "name of biogas plant _sampling date (the last two numbers of year/month)_digester ID in the plant", while "CZ_0d" and "LM_0d" represent the inoculating microbiota. (c) PCoA of microbial community structure (based on 16S rRNA gene amplicon data) of full-scale anaerobic digesters where the inoculating microbiota come from, using unweighted UniFrac distance matrix.
