# Supplementary material for: Syntrophic Acetate-Oxidizing Microbial Consortia Enriched from Full-Scale Mesophilic Food Waste Anaerobic Digesters Showing High Biodiversity and Functional Redundancy
Source: mSystems. 2022 Sep 8;7(5):e00339-22. doi: 10.1128/msystems.00339-22 (PMC9600251; doi:10.1128/msystems.00339-22)
Supplement: FIG S5 [file msystems.00339-22-s0005.pdf]

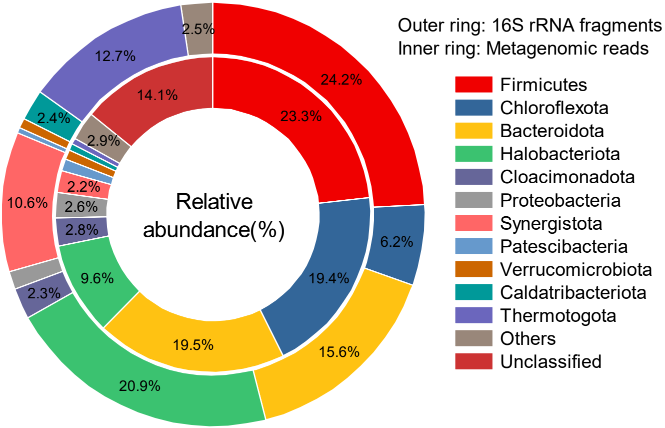

Figure S5. Comparison of microbial composition based on taxonomic annotation of MAGs (inner ring) and 16S rRNA gene amplicons (outer ring) on the phylum level for the six samples.
