# Supplementary figures and images for: Syntrophic Acetate-Oxidizing Microbial Consortia Enriched from Full-Scale Mesophilic Food Waste Anaerobic Digesters Showing High Biodiversity and Functional Redundancy
Source: mSystems. 2022 Sep 8;7(5):e00339-22. doi: 10.1128/msystems.00339-22 (PMC9600251; doi:10.1128/msystems.00339-22)

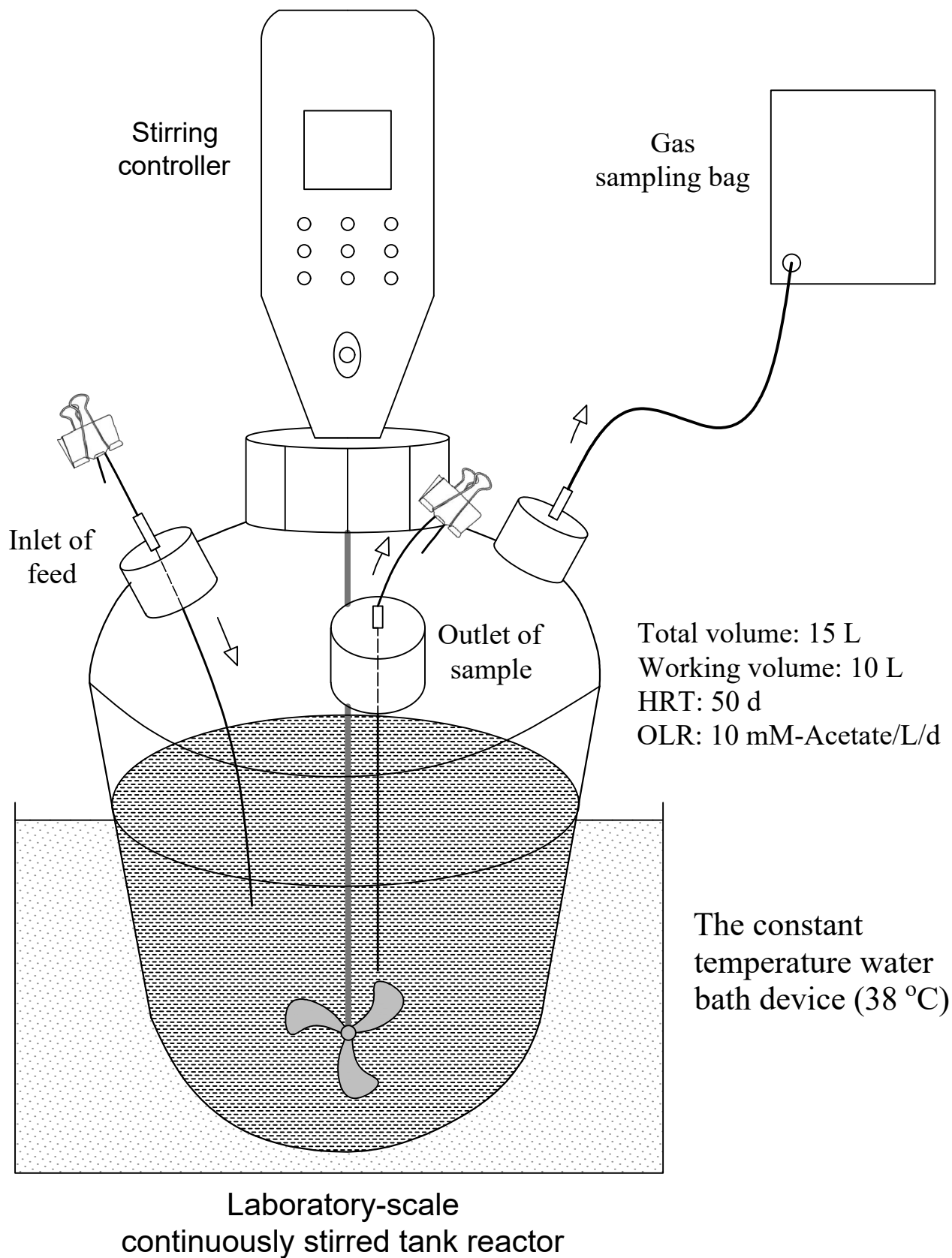

**Figure S6.** Schematic of the laboratory reactor (continuous stirring at 150 rpm)

Supplement: FIG S6 [file msystems.00339-22-s0006.pdf]
