# Supplementary material for: Syntrophic Acetate-Oxidizing Microbial Consortia Enriched from Full-Scale Mesophilic Food Waste Anaerobic Digesters Showing High Biodiversity and Functional Redundancy
Source: mSystems. 2022 Sep 8;7(5):e00339-22. doi: 10.1128/msystems.00339-22 (PMC9600251; doi:10.1128/msystems.00339-22)
Supplement: TEXT S1 [file msystems.00339-22-s0010.docx]

# **Supplementary Text**

**Text S1.** **Details of the experimental procedure of metagenomic sequencing**

1 Sample testing

Two methods were used in quality control of DNA samples: (1) DNA quality was monitored on 1% agarose gels. (2) DNA concentration was measured using Qubit® dsDNA Assay Kit in Qubit® 2.0 Flurometer (Life Technologies, CA, USA).

OD value is between 1.8~2.0, DNA contents above 1 µg are used to construct the sequencing library.

2 Library construction

A total amount of 1 µg DNA per sample was used as input material for the metagenomics sequencing library preparations. Sequencing libraries were generated using NEBNext® Ultra^TM^ DNA Library Prep Kit for Illumina (NEB, USA) following manufacturer’s recommendations and index codes were added to attribute sequences to each sample. Briefly, the DNA sequences were fragmented by sonication to a size of 350 bp, then DNA fragments were end-polished, A-tailed, and ligated with the full-length adaptor for Illumina sequencing with further PCR amplification. At last, PCR products were purified (AMPure XP system) and libraries were analyzed for size distribution by Agilent2100 Bioanalyzer and quantified using real-time PCR.

3 Sequencing

The clustering of the index-coded samples was performed on a cBot Cluster Gencration System according to the manufacturer's instructions. After cluster generation, the prepared libraries were sequenced on an Illumina HiSeq platform and paired-end reads were generated.
